# Differential Mitochondrial Genome Expression of Four Hylid Frog Species under Low-Temperature Stress and Its Relationship with Amphibian Temperature Adaptation

**DOI:** 10.3390/ijms25115967

**Published:** 2024-05-29

**Authors:** Yue-Huan Hong, Ya-Ni Yuan, Ke Li, Kenneth B. Storey, Jia-Yong Zhang, Shu-Sheng Zhang, Dan-Na Yu

**Affiliations:** 1 College of Life Sciences, Zhejiang Normal University, Jinhua 321004, China; 2 Department of Biology, Carleton University, Ottawa, ON K1S 5B6, Canada; 3 Key Lab of Wildlife Biotechnology, Conservation and Utilization of Zhejiang Province, Zhejiang Normal University, Jinhua 321004, China

**Keywords:** Hylidae, temperature stress, RT-qPCR, mitochondrial gene expression

## Abstract

Extreme weather poses huge challenges for animals that must adapt to wide variations in environmental temperature and, in many cases, it can lead to the local extirpation of populations or even the extinction of an entire species. Previous studies have found that one element of amphibian adaptation to environmental stress involves changes in mitochondrial gene expression at low temperatures. However, to date, comparative studies of gene expression in organisms living at extreme temperatures have focused mainly on nuclear genes. This study sequenced the complete mitochondrial genomes of five Asian hylid frog species: *Dryophytes japonicus*, *D. immaculata*, *Hyla annectans*, *H. chinensis* and *H. zhaopingensis*. It compared the phylogenetic relationships within the Hylidae family and explored the association between mitochondrial gene expression and evolutionary adaptations to cold stress. The present results showed that in *D. immaculata*, transcript levels of 12 out of 13 mitochondria genes were significantly reduced under cold exposure (*p* < 0.05); hence, we put forward the conjecture that *D*. *immaculata* adapts by entering a hibernation state at low temperature. In *H. annectans*, the transcripts of 10 genes (*ND1*, *ND2*, *ND3*, *ND4*, *ND4L*, *ND5*, *ND6*, *COX1*, *COX2* and *ATP8*) were significantly reduced in response to cold exposure, and five mitochondrial genes in *H. chinensis* (*ND1*, *ND2*, *ND3*, *ND4L* and *ATP6*) also showed significantly reduced expression and transcript levels under cold conditions. By contrast, transcript levels of *ND2* and *ATP6* in *H*. *zhaopingensis* were significantly increased at low temperatures, possibly related to the narrow distribution of this species primarily at low latitudes. Indeed, *H. zhaopingensis* has little ability to adapt to low temperature (4 °C), or maybe to enter into hibernation, and it shows metabolic disorder in the cold. The present study demonstrates that the regulatory trend of mitochondrial gene expression in amphibians is correlated with their ability to adapt to variable climates in extreme environments. These results can predict which species are more likely to undergo extirpation or extinction with climate change and, thereby, provide new ideas for the study of species extinction in highly variable winter climates.

## 1. Introduction

Hylidae (Anura, Neobatrachia) is an abundant family of amphibians, with 885 species in 57 genera [1,2]. Although commonly known as tree frogs, many species are also found in other environments such as in rice fields, reed marshes and other wetlands. Hylidae has three subfamilies: Hylinae, Phyllomedusinae and Pelodryadinae. Hylinae is the subfamily containing the most frogs and all eight species found in China belong to this subfamily. These are *Hyla annectans*, *H*. *chinensis*, *H*. *sanchiangensis*, *H*. *simplex*, *H*. *tsinlingensis*, *H*. *zhaopingensis*, *Dryophytes immaculata* and *D*. *japonica* (https://amphibiansoftheworld.amnh.org/, accessed on 20 June 2023) [3] and (http://www.amphibiachina.org/, accessed on 20 June 2023) [4]. Four species are included in the present study. *D. immaculata* (Boettger, 1888) [5] (Anura: Hylidae) is known to range from Guangdong in the south to Heibei. *H*. *annectans* (Jerdon, 1870) [6] (Anura: Hylidae) is mainly distributed in western China (Yunnan, Guizhou, Sichuan, Hunan), but also in India, Myanmar and Thailand. *H*. *chinensis* (Günther, 1858) [7] is found mainly in southern China, but also in northern Vietnam, and *H*. *zhaopingensis* (Tang and Zhang, 1984) [8] (Anura: Hylidae) was discovered at Zhaoping of Guangxi, China.

Previous studies focused on the species boundary between *D. suweonensis* and *D. japonica* [9,10]. However, Li et al. [11] proposed that the sequences of *D. immaculata* and *D. suweonensis* obviously clustered together in the phylogenetic tree to form a homologous monophyletic branch, but the *D. suweonensis* sequence adopted in that study lacked detailed information about sampling points. Dufresnes [12] also supported the synonym relationship between *D. immaculata* and *D. suweonensis*. However, the results of Borzee et al. [13] highlighted differences and divergences between the clades of *D. suweonensis* and *D. immaculata*, and suggested that *D. suweonensis* occurred in a ring around the Yellow Sea via continuous genetic variation of haplotypes, but this was difficult to prove. The same study also showed differences between *D. suweonensis* and *D. immaculata* based on morphology and acoustics in the latter study [14]. Hence, at present, the question of whether these two are the same species or not needs further experimentation.

Global climate change has exerted extensive and profound impacts on species, populations and ecosystems [15,16,17]. In recent years, short extreme cold weather events in mainland China have also become more frequent. More and more evidence suggests that the global climate is changing, and a biological response to climate change is ongoing [18]. Many species can adapt and survive the effects of climate change, but the current rate of temperature rise is unprecedented and faster than previous climate change events [18]. This means that extreme heat and cold events will increase in the future. In addition, if a population of one species cannot adapt to new environmental conditions brought about by climate change, this may lead to species extirpation from local areas or even to full extinction, posing a serious threat to biodiversity [19]. The unstable weather caused by global warming has resulted in a drastic decline for several species [20], with a variety of population declines and extinctions being reported in recent decades [21,22,23,24]. Scientists predict that species may respond to these global events by changing their behavior and altering their range [25]. Therefore, whether organisms can survive extreme temperatures and initiate a series of regulatory mechanisms becomes a crucial issue.

Compared with mammals and birds, amphibians are more readily affected by climate change. Indeed, the study of amphibians for climate change is of particular importance in the context of global climate change because they are very sensitive to environmental stresses [26,27]. Hence, they are considered to be good models for seeking out the factors leading to genetic variation and differentiation patterns [28,29]. In particular, amphibians are subject to dual pressures because they have both aquatic and terrestrial phases in their life [30]. Moreover, compared to homeothermic animals, poikilotherm animals are more likely to track changes in their climatic space [31]. Studying the relationship between animal species distribution and climate variables is crucial for improving our ability to predict the ecological consequences of future climate change [32,33,34]. Due to the high sensitivity of poikilotherms to temperature change, amphibians regulate their metabolism and several species activate a series of cold resistance mechanisms under low-temperature stress [34]. Indeed, the use of liver glycogen to produce cryoprotectants at the onset of freezing occurs in several frost-tolerant amphibians [35,36,37,38,39,40]. As a species that can live at 0 °C (or lower) for a long time, *R. sylvatica* can also endure prolonged time under hypoxic/anoxic conditions [41]. Indeed, many organisms that are subjected to low-temperature stress show a strong suppression of metabolic rate [42,43,44]. For example, the resting metabolic rate of the toad *Bufo marinus* temporarily decreases under low-temperature exposure [45]. In addition, mtDNA has a variety of unique properties that enable it to act as a cellular sentinel for genotoxic stress [46], responding to temperature extremes ahead of nuclear genes. When amphibians are exposed to low-temperature stress, the expression of cold-resistance-related genes can be activated and regulated along with the synthesis of stress-related substances in order to reduce damage to their bodies [35,36,37,38,39,40,41,42,43,44,45,46]. Furthermore, since antioxidant defenses are closely related to cold tolerance, the transcription levels of mitochondrial protein-coding genes, which are closely related to ATP generation, can also reflect the expression of cold-resistance-related genes.

Mitochondria play an important role in the adaptation of organisms to environmental temperature change and are closely linked to oxygen restriction [47,48,49,50,51,52,53]. Mitogenomes are also currently used as effective tools for species identification and for determining phylogenetic relationships [54]. Although there have been many studies of anuran mitogenomes in order to elucidate phylogenetic relationships, few studies have included data on mitochondrial gene expression, and most have focused on the expression of nuclear genes. However, in recent years, some data have been gathered to describe mitochondrial gene expression responses to environmental stress (e.g., temperature). For example, Zhang et al. [54] showed that expression of the mitochondrial *COX1* gene in *D*. *versicolor* was significantly reduced at low temperature. Jin et al. [55] conducted cold stress experiments in two different populations of *Hoplobatrachus rugulosus*. The results showed that Thai *Ho. rugulosus* (TT frogs) showed a fast growth rate and high-temperature tolerance, whereas Chinese *Ho. rugulosus* (CT frogs) had a slower growth rate and a strong tolerance of low temperature [47]. Hence, mitochondrial gene expression varies both by species and by the stress encountered.

Li et al. [11] showed that the genus *Hyla* originated in North America and then spread to China across the Bering land bridge during the Middle Eocene to Early Oligocene. Hence, *Dryophytes* and *Hyla* have been separated from other Hylidae for about 22.6 mya [56]. The Hylidae family spread from north to south [11]. However, Yan et al. [57] argued that the *Hyla chinensis* group originated in southern and eastern China. Since *Dryophytes* and *Hyla* used to be considered as one genus, Dullman et al. proposed that *Dryophytes* should be a separate genus [58]. The selection of these two genera as subjects also helped to find differences between them.

To further explore the mechanisms of amphibian response to low temperature, we selected *Dryophytes* and *Hyla* as the research targets for a study of mitochondrial gene expression. Liver is a main organ of energy metabolism in organisms [59], and studies have proven that using liver as a research model can obtain significant expression results [55]. Therefore, in this study, liver was selected as the main organ with which to explore the situation of mitochondrial gene expression under the stress of low temperature. In addition, studying gene expression of amphibians at low temperatures can help us to understand the tolerance of individual species for extreme temperatures. The least tolerant species are more likely to go extinct in the context of frequent temperature extremes, which also provides new ideas for the study of species extinction in extreme climates.

## 2. Results

### 2.1. General Features of the Mitogenome

We obtained the nearly complete mitochondrial genomes of five Hylidae species (except for part of the control region), namely, *D*. *japonicus*, *D*. *immaculatus*, *H*. *annectans*, *H*. *chinensis* and *H*. *zhaopingensis*. These mitogenomes were loaded into GenBank with identification numbers OR398492, OR398491, OR398488, OR398389 and OR398490, respectively, and gene lengths of 17,221 bp, 18,186 bp, 17,060 bp, 17,087 bp and 15,812 bp, respectively. Gene arrangements were similar to those of other hylid species. Figure S1 shows the mitochondrial gene arrangement order in Hylidae.

The location and characteristics of each gene are shown in Table S1. Of the five mitochondrial genomes of Hylidae assessed in this study, four of them used ATN as the start codon, whereas the *ND1* gene used TTG. Most stop codons were full stop codons TAN and AGA, whereas an incomplete stop codon T appeared in *ND1*, *ND3* and *COX2*. In addition, TA terminators were found on all *COX3* genes. The 12S rRNA was located between trnF and trnV, with a length of 932~938 bp, whereas 16S rRNA was located between trnV and trnL2, with a length of 1595~1601 bp. The mitochondrial genomes of the five species of Hylidae had non-coding regions and overlapping regions. The longest overlapping region appeared between *ATP6* and *ATP8*, with a length of about 10 bp, and the longest interval region appeared between trnS1 and *ND5*, with a length of about 35 bp. And the five species of Hylidae all showed the typical characteristics of vertebrates, with an obvious AT skew. AT content and AT skew data are shown in Table 1.

**Figure 1:**
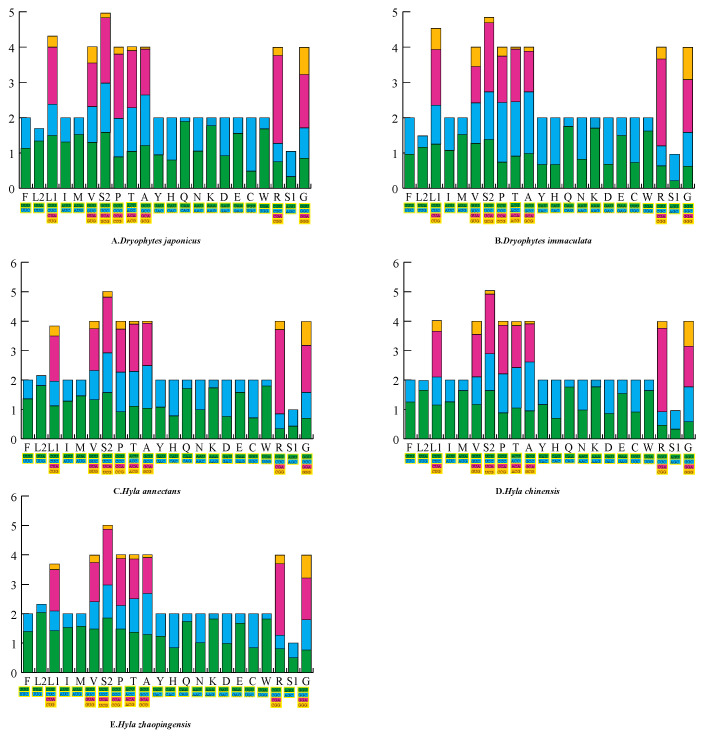
Relative synonymous codon use (RSCU) of (**A**) *Dryophytes japonicus*, (**B**) *Dryophytes immaculata*, (**C**) *Hyla annectans*, (**D**) *Hyla chinensis* and (**E**) *Hyla zhaopingensis*. All the codons used, as well as different combinations of synonymous codons, are listed on the *X*-axis, whereas the RSCU values are listed on the *Y*-axis. Different codons are represented by different colors.

The RSCU is shown in Figure 1, and the original data are found in Supplemental Table S2. The results showed that each codon had a different frequency of use in the genome and that Hylidae species showed preferential use of A and T in synonymous codons. CGA in arginine (Arg) was the most frequently used codon among all amino acids. In addition, UCA in serine (Ser2) was also frequently used, whereas GCG in alanine (Ala) was the least frequently used amino acid.

**Figure 2:**
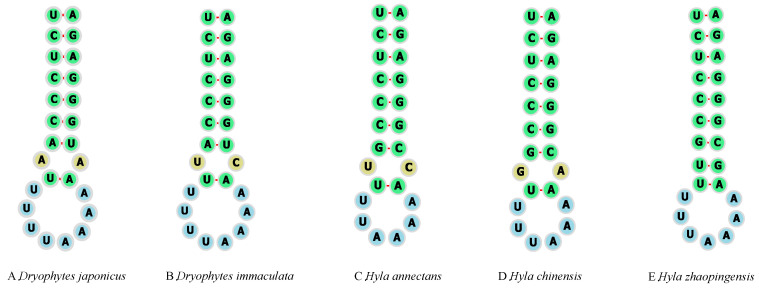
The secondary structures for L-strand origin of replication (O_L_) for individuals of (**A**) *Dryophytes japonicus*, (**B**) *Dryophytes immaculata*, (**C**) *Hyla annectans*, (**D**) *Hyla chinensis* and (**E**) *Hyla zhaopingensis*.

The tRNA secondary structures of the five sequences of Hylidae species are shown in Figures S2–S6, respectively. The tRNA length was similar for all species, and the secondary structure was the typical clover leaf. There were base pair mismatches in some tRNAs, the most common being U-G mismatches. In addition, a starting region called the L-strand origin (O_L_) for replication was found between trnN and trnC and it plays an important role in replication. The O_L_ between trnN and trnC was approximately 25 bases with a stem ring structure (Figure 2). Mismatch was found in the O_L_ of the Hylidae in this study. There was a U-G mismatch in *H*. *zhaopingensis*. Moreover, the numbers of U bases and A bases in the stem rings of the five Hylidae were different, and the lengths of the stem loops were also different.

### 2.2. Genetic Distance and Phylogenetic Relationships

**Figure 3:**
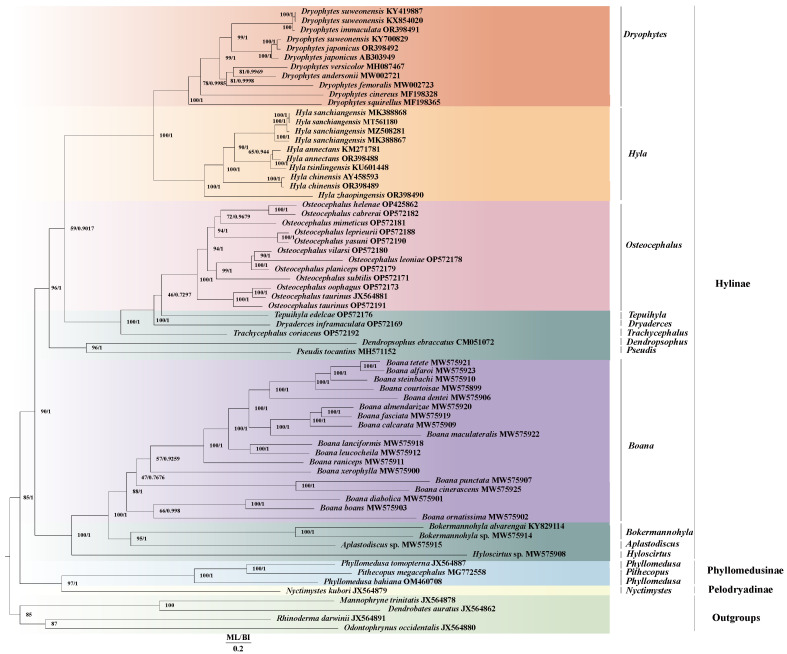
First, second and third positions of codon conformational tree results. BI and ML analyses were used to predict the phylogenetic relationships among the Hylidae based on the nucleotide data set encoding the 13 proteins. Species name information and GenBank number are marked on the figure. Posterior probabilities (PPs) of BI and Bootstrap values (BPs) of ML analyses are shown at the nodes.

The tree results of first-, second- and third-position codon constructions are shown in Figure 3, and the tree results of the first- and second-position constructions are shown in Figures S7 and S8. We found higher confidence levels (CLs) in results using first-, second- and third-position conformational trees, and so these data were used in this study. The results from the construction of the phylogenetic trees showed that this study included three subfamilies of Hylidae, including Hylinae, Phyllomedusinae and Pelodryadinae. The results showed that Pelodryadinae and Phylomedusinae converged into one branch, and then converged with Hylidae. The phylogenetic relationship of ((((((((((*Dryophytes* + *Hyla*) + *Osteocephalus*) + *Dryaderces*) + *Tepuihyla*) + *Trachycephalus*) + (*Dendropsophus* + *Pseudois*)) + *Boana*) + *Bokermannohyla*) + *Aplastofdiscus*) + *Hyloscirtus*) appeared in the Hylinae subfamily. This research supported Dullman’s findings [58] that *Hyla* and *Dryophytes* were monophyletic, and the monophyletic nature of the Hylidae was also supported in this study.

In order to resolve the dispute about the phylogenetic relationships of *D*. *suweonensis*, *D*. *japonicus* and *D*. *immaculata*, we analyzed the genetic distance of all relevant sequences on the NCBI (accessed on 20 June 2023) as well as the two sequences for *D*. *japonicus* and *D*. *immaculata* reported in this study. The results shown in Table 2 indicate that the genetic distance between *D*. *suweonensis* (KY700829) and the other two *D*. *suweonensis* samples (KX54020 and KY419887) [60,61] was relatively large at 12.2%, and was closer to the *D*. *japonicus* sequences reported earlier (AB303949) [62] and reconfirmed in this study. In addition, the phylogenetic tree (Figure 3) showed that the sequences of *D*. *suweonensis* (KY700829) and two *D*. *japonicus* species were clustered together, which may be due to an error in the identification of the sequence of *D*. *suweonensis* (KY700829).

### 2.3. Effect of Cold Exposure on Transcript Levels of PCGs

**Figure 4:**
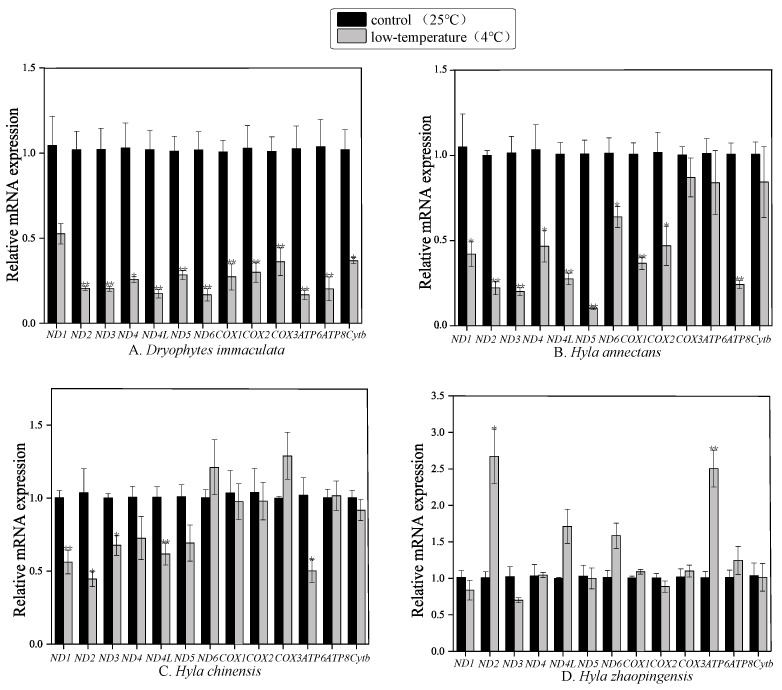
Relative expression of mitochondrial genes under control (25 °C) and low-temperature (4 °C) stress in (**A**) *Dryophytes immaculata*, (**B**) *Hyla annectans*, (**C**) *Hyla chinensis* and (**D**) *Hyla zhaopingensis*, where “*” indicates a significant difference (*p* < 0.05) and “**” indicates (*p* < 0.01).

Relative transcript levels of the 13 mitochondrial protein-coding genes from the four species of Hylidae were obtained from liver samples and analyzed by RT-qPCR to compare mRNA levels in liver of control frogs held at 25 °C with frogs transferred to 4 °C for 24 h (hypothermia group) (Figure 4). The original data are shown in Table S3. The results showed that, compared with the control group (24 °C), mitochondrial gene expression in liver of the four Hylidae species was significantly altered in response to 24 h cold exposure at 4 °C. 

Transcript levels of two genes in *H*. *zhaopingensis* liver (*ND2* and *ATP6*) were significantly elevated (*p* < 0.05) in response to cold exposure by 2.67 ± 0.37 and 2.50 ± 0.25-fold, respectively (Figure 4D). In contrast, transcript levels in liver of *D*. *immaculata*, *H*. *annectans* and *H*. *chinensis* were unchanged or significantly reduced.

In *D*. *immaculata*, transcript levels of 12 out of 13 mitochondria genes were significantly reduced under cold exposure (*p* < 0.05). Transcript levels of *ND2*, *ND3*, *ND4*, *ND4L*, *ND5*, *ND6*, *COX1*, *COX2*, *COX3*, *ATP6*, *ATP8* and *Cytb* were reduced by 0.21 ± 0.01, 0.20 ± 0.02, 0.26 ± 0.02, 0.18 ± 0.03, 0.29 ± 0.03, 0.17 ± 0.04, 0.27 ± 0.08, 0.30 ± 0.06, 0.36 ± 0.08, 0.17 ± 0.03, 0.20 ± 0.07 and 0.37 ± 0.02-fold, respectively, as compared with controls.

In *H*. *annectans* liver transcripts of 10 genes (*ND1, ND2, ND3, ND4, ND4L, ND5, ND6, COX1, COX2* and *ATP8*) were significantly decreased in response to cold exposure, with reductions by 0.42 ± 0.07, 0.22 ± 0.04, 0.20 ± 0.02, 0.47 ± 0.09, 0.28 ± 0.03, 0.10 ± 0.01, 0.64 ± 0.06, 0.37 ± 0.04, 0.47 ± 0.12 and 0.24 ± 0.02-fold, respectively, as compared with controls.

Cold exposure at 4 °C had a lesser effect on mitochondrial gene expression in *H. chinensis.* Five mitochondrial genes showed significantly reduced expression. Transcript levels of *ND1, ND2, ND3, ND4L* and *ATP6* were reduced by 0.56 ± 0.08, 0.45 ± 0.05, 0.68 ± 0.07, 0.62 ± 0.08 and 0.50 ± 0.08-fold, respectively, as compared with controls.

## 3. Discussion

### 3.1. Mitogenome Structure, Genetic Distance and Phylogeny of Hylidae

A non-coding region with a length of about 35 bp was found between trnS1 and *ND5* in the five Hylidae sequences evaluated in this study. This non-coding region has been found in many species of Hylidae [62,63,64,65,66,67,68], but did not exist in *D. versicolor* [54]. Whether this is the feature of all members of Hylidae will require more sequences to be assessed. Since *D. versicolor* is a North American species, the absence of this non-coding region might suggest a characteristic unique to Asian species of Hylidae. In the previous concept, the non-coding region was considered to be non-functional, but the current study has made progress in annotating these non-coding regions, and the function of this non-coding region needs further research [69].

The genetic distance between *D*. *immaculata* and *D*. *suweonensis* (KY419887 and KX854020) [60,61] in this study was only 0.8%, and the two species also converged into one branch on the phylogenetic tree. Therefore, at the molecular level, it appeared that the relationship between the two species appeared very close, which was consistent with previous results [54]. However, due to a lack of morphological and other information on *D. suweonensis*, further research is needed.

### 3.2. Different mt Gene Expression between Different Species of Dryophytes and Hyla

In *D*. *immaculata*, *H*. *annectans* and *H*. *chinensis*, all genes that showed significant differences were reductions in expression in response to low-temperature exposure. However, in *H*. *zhaopingensis*, expression of both *ND2* and *ATP6* genes was strongly and significantly increased, with an upward trend also seen for *ND4L* and *ND6*. Previous studies focused mainly on the physiology of frogs, but in fact, temperature-adaptive transformation of gene expression is a common mechanism of physiological adaptation, particularly for seasonal adaptation to changing environmental temperatures [70]. Due to the close correlation between antioxidant defense and freeze tolerance [47], protein-coding gene expression in the different complexes of mitochondria, which are metabolic energy centers, will very likely change when poikilothermic animals need to adjust to a low-temperature environment. Hence, down-regulation of mitochondrial activity is a simple way to promote entrance into a dormancy by establishing a low-energy metabolic pathway and reducing the demand for ATP, so as to preserve the fuel required for long-term survival in low-temperature environments, particularly when food sources are unavailable during the winter season [71]. This inhibition is likely also the reason for the significant decrease in the expression levels of multiple genes in mitochondria.

We found that only the *ND2* gene was significantly different in its expression pattern among the four Hylidae, and in the three species with reduced *ND2* expression, *ND3* and *ND5* gene expression was also significantly decreased. In fact, there are many more species that show genetic differences in *ND* gene expression in response to various stresses. The proteins encoded by these genes are concentrated in mitochondrial complex I, and positive selection sites in previous studies were also concentrated mostly in mitochondrial complex I [65]. Mitochondrial complex I is a large enzyme and is the main entry point for electrons delivered from nicotinamide adenine dinucleotide (NADH) into the respiratory chain [72]. Complex I oxidizes NADH, transfers electrons to ubiquinone (CoQ) and is generally considered to be the site of the main reactive oxygen species (ROS) producing enzyme in mitochondria, which is closely related to energy production [73]. Not surprisingly, the strong response of the ND series of genes is closely related to a change in mitochondrial energy metabolism.

With the exception of *H*. *zhaopingensis*, the Hylidae family members analyzed in this study all showed a downward trend in mitochondrial gene expression under 4 °C low-temperature stress. That is, reduced demand for ATP lowers metabolic rate so as to prolong survival time. Among these genes, the most responsive changes in expression occurred in *D*. *immaculata* with the expression of 12 out of 13 mitochondrial-encoded proteins showing strong and significant down-regulation in response to decreased temperature. Compared with *H*. *annectans* and *H*. *chinensis*, and except for the ND series genes, the expression of all COX series, ATP genes and *Cytb* genes in *D*. *immaculata* were also significantly reduced in response to low temperature. COX series genes are located in cytochrome c oxidase (Complex IV), the last and rate-limiting step in the respiratory chain and closely related to prevention of the formation of ROS [74]. The ATP series genes are located in ATP synthase (Complex V), which is a key enzyme in cell respiration [75]. *Cytb* is located in mitochondrial complex III and catalyzes the transfer of electrons from succinic acid and nicotinamide adenine dinucleotide-linked dehydrogenase to mitochondrial-encoded cytochrome b [76]. In addition, the ND series genes located on complex I regulate the oxidation of NADH. The decrease in expression levels of these genes directly affects multiple links of the respiratory chain and can lead to reduced activity of related respiratory chain enzymes, blockage of mitochondrial electron transport and the activity of electron transfer6 [77]. Hence, a down-regulation of mitochondrial gene expression can lead to a decrease in ATP production, leading to less harmful reactive oxygen species that help organisms to combat cold environments.

Among various species, there are generally significant differences in thermal tolerance limits and the ability to regulate these limits in a temperature-adaptive manner among species living in variable temperatures [69]. Including *H*. *zhaopingensis* in this study, we also showed that the expression of mitochondrial-encoded proteins increased under low-temperature stress. These increases in mitochondrial gene expression in *H*. *zhaopingensis* mainly affected mitochondrial respiratory chain complexes I and V that include the NADH dehydrogenases and ATP synthase, thus increasing the expression of key enzymes of the respiratory chain complex. This phenomenon is similar to the principle of increased mitochondrial expression in some species. For example, transcription of the mitochondrial genes *ATP6*/*8*, *ND4* and 16S RNA in the freeze-tolerant wood frog, *Rana sylvatica,* was strongly up-regulated in liver and brain during whole body freezing (−2.5 °C, 24 h) [47], and the expression of *ATP6* and *ATP8* in mitochondria of tilapia fish, *Oreochromis aureus*, increased at 12 °C compared with that at 24 °C [78]. This also shows that species from different regions may adapt to different temperatures and/or have different adaptability to the same temperature.

### 3.3. The Relationship between Mitochondrial Gene Expression and Temperature Adaptation

According to the above theory, we chose *D*. *immaculata* as the most suitable species for low-temperature studies among the four frog species for three reasons. The first was that, compared to the *Hyla* genus, the *Dryophytes* genus is distributed at higher latitudes and these frogs live in colder environments. Bozinovic et al. [79] proposed that the tolerance range of organisms is related to their phenotypic flexibility, so the physiological flexibility of individuals, species and populations should increase with latitude. Therefore, *D*. *immaculata* was more likely to be adaptable to low-temperature stress. After prolonged exposure to low temperature, this species can also quickly adjust to temperature changes and actively reduce energy consumption, thus obtaining a longer survival time. The second reason was related to the distribution range, that is, the adaptation of organisms to a long-term living environment. *D*. *immaculata* is widely distributed in China, ranging from Guangdong in the south to Heibei in the north. By contrast, *Hyla chinensis* is mainly distributed in southern China, *H*. *annectans* is mainly distributed in the southwest and *H*. *zhaopingensis* is distributed in Zhaoping, Guangxi (http://www.amphibiachina.org/, accessed on 20 June 2023). Overall, this indicates that *D*. *immaculata* is a more adaptable frog that can endure different temperatures. Finally, according to the phylogenetic results for *H*. *zhaopingensis*, which is located at the base of the genus, and the research by Li et al. [11] and Yan et al. [57], we hypothesized that the *Dryophytes* genus originated in the north and spread southward, whereas the *Hyla* genus originated in the south and spread north. This also appears to be why *D*. *immaculata* is better adapted to low temperatures.

The climate variability hypothesis assumes that tropical organisms should have lower physiological plasticity due to the reduced thermal variability in which they evolved and live [80,81,82]. In line with this theory, temperate species exhibit tolerance over a wider temperature range and have a larger thermal safety threshold than tropical species [83,84]. This is also the reason why the species of *H*. *zhaopingensis* located at low latitudes are the least adapted to low temperatures. The monthly average temperature in the winter (December to February) from 1971 to 2010 in the region where *H*. *zhaopingensis* lives is higher than 8 °C (China National Climate Center, http://cmdp.ncc-cma.net/cn/index.htm, accessed on 22 June 2023). In addition, *Hyla zhaopingensis* has the narrowest distribution range among the four species [11]. Such a narrow distribution range can also lead to an inability to adapt to low temperatures. For all of the above reasons, *H*. *zhaopingensis* shows little or no ability to adapt to low temperatures, such as 4 °C, and cannot enter a hibernation state. Instead, when faced with cold temperatures, this species entered a state of metabolic disorder. The mitochondria of this species cannot enter low-energy metabolic pathways, but instead attempts are made to restore the frog to a normal temperature by increasing the expression of encoded proteins and increasing ATP production to generate more heat. Indeed, if the temperature falls below 0 °C, ice crystals can form in the frog’s body, causing physical damage to cells, subcellular structure and the compartmentation of subcellular organelles, eventually leading to its death [47,85].

The present study found significant differences in gene expression and cold tolerance mechanisms among the different species of Hylidae under the same temperature stress, indicating that there were differences in the temperature tolerance range and cold tolerance of these four species. The results of this study indicate that *H*. *zhaopingensis* is the most ill-adapted species for low-temperature conditions among the four species studied and is also the species that is predictably the most susceptible to extinction under extreme environmental temperature change. In addition, because of its narrow distribution and low biodiversity, *H*. *zhaopingensis* could become extinct if extreme low temperature caused the death of a large number of individuals. Therefore, the study of mitochondrial genome expression changes under low-temperature stress can be used as a monitoring method to determine whether species are vulnerable to extinction, as well as to provide ideas for amphibian diversity conservation. In addition, transcriptome technology has gradually developed over the past two years, and by studying the transcriptome’s response to temperature stress, we can better understand the genes and biochemical pathways that are critical for physiological adaptation to a warmer environment and gain insight into the regulatory changes that accompany adaptation on evolutionary timescales [86].

## 4. Materials and Methods

### 4.1. Sample Collection and Cold-Stress Treatment

Samples of *D. immaculata* were collected from Chuzhou, Anhui, China (24.14° N, 110.18° E), samples of *H. annectans* were from Anshun, Guizhou, China (26.24° N, 105.93° E), samples of *H. chinensis* were collected from Suzhou, Jiangsu, China (31.30° N, 120.62° E) and samples of *H. zhaopingensis* were from Maoming, Guangdong, China (21.64° N, 110.91° E). All frogs were collected in July 2022, with 20 frogs collected at each location. The samples for the same species consisted of male frogs with similar body sizes. All animals were washed in a tetracycline bath and placed in a plastic incubator (90 cm × 40 cm × 60 cm) at 25 °C for one week. Ten from each group were randomly selected from the 25 °C temperature group and placed in a plastic box under a wet towel at 25 °C for 24 h as the control group. At the same time, ten frogs were subjected to 4 °C hypothermia stress for 24 h as the hypothermia stress group. In this study, rapid freezing was used, that is, frogs at 25 °C were directly exposed to 4 °C without undergoing a slow cooling process. Subsequently, the control group and the hypothermic temperature group were euthanized by pithing; then, the livers were rapidly dissected and frozen in liquid nitrogen. Subsequently, liver samples were stored in an ultra-low-temperature freezer at −80 °C until use. In addition, this study also included sequencing of the *D. japonicus* mitogenome.

### 4.2. Total DNA Extraction, Primer Design, PCR Amplification and Sequencing

Tissue samples were obtained from a clipped toe of each specimen and stored in 100% ethanol at −40 °C for subsequent DNA extraction. Total genomic DNA was extracted using the Ezup Column Animal Genomic DNA Purification Kit (Sangon Biotechnology, Shanghai, China) according to the manufacturer’s manual. The methods used for DNA extraction and PCR amplification are as described by Cai et al. [87] and the general primers used in this study were as described by Zhang et al. [88]. Sequence proofreading was then performed, and specific primers were designed using Primer Premier 5.0 (Primer Biosoft International, San Francisco, CA, USA) based on the fragments measured by the universal primers. To further identify the species and ensure that each quantitative sample for mitochondrial gene expression was the same species with low gene distance difference, DNA was extracted from the toe of all samples and the *COX1* gene was amplified by PCR. All PCR products were purified and sequenced by Sangon Biotechnology (Shanghai, China). Nei proposed in 1971 and 1972 that the genetic distance between genes (D = −log_e_^I^) could be used to measure genetic differences between different populations [89], and this method has been widely used in species identification, population classification and genetic correlation analysis of species [90]. Results obtained from the genetic distance method for *COX1* are shown in Table S4. Samples with genetic distance less than 1% were selected for quantitative experiments.

### 4.3. Mitogenome Annotation and Sequence Analyses

Seqman in DNASTAR v.6.0 was used for splicing the sequencing results from the four species and Sanger sequencing was manually checked and assembled [91]. All tRNA genes were annotated using MITOS2 (http://mitos2.bioinf.uni-leipzig.de/index.py, accessed on 20 June 2023) [92]. We used MEGA 11.0 [93] to identify and annotate 12S rRNA, 16S rRNA and 13 protein-coding genes (PCGS), and we compared their homology. The tRNAscan—SE1.21 program [94] (http://lowelab.ucsc.edu/tRNAscan-SE/, accessed on 20 June 2023) and the MITOS2 program [92] were used to predict the cloverleaf secondary structure of all tRNA genes. RNAalifold (http://rna.tbi.univie.ac.at/cgi-bin/RNAWebSuite/RNAalifold.cgi, accessed on 20 June 2023) was used to draw all tRNA genes of the replication origin region and secondary structure [95]. PhyloSuite [96] was used to identify the 13 protein-coding genes and calculate relative synonymous codon usage (RSCU) and AT content, and AT skewness was calculated using AT skew = (A − T)/(A + T) and GC skew = (G − C)/(G + C) [97].

### 4.4. Genetic Distance and Phylogenetic Analyses

Using MEGA11 [66], the genetic distances of all data available for *D. immaculata*, *D. suweonensis* and *D. japonicus* in the NCBI and the experimental samples were calculated using the Kimura 2-parameter model [98]. We downloaded 63 mitogenomes of the family Hylidae from the NCBI to study the relationships between our five sequences and other species of the family Hylidae. Table S8 shows GenBank numbers for the 68 species that were used to construct phylogenetic trees. The 13 protein-coding genes were extracted in PhyloSuite [96] for MAFFT [99] comparison, and conserved regions were selected using Gblock [100] and then linked together using concatenate sequence in PhyloSuite [96]. DAMBE [101] was used to analyze the saturation of the third-codon positions. The results are shown in Table S5. This study used first-, second-, and third-codon positions as well as the first- and second-codon positions for tree construction since the third-codon position had a slightly saturated state. The optimal partition and evolutionary model were selected using the Bayesian information criterion (BIC) [102] in PartitionFinder v2.1.1 [103] (Tables S6 and S7). The partition results were used for Bayesian inference (BI) analysis in MrBayes version 3.2 [104], and the posterior probability (PP) was calculated mainly via the Markov Chain Monte Carlo method (MCMC). Starting from the random tree, 10 million generations were run, and samples were taken every 1000 generations. Based on convergence (<0.01), the first 25% of runs were discarded as aging. The remainder was used to construct the BI phylogenetic tree. The partition result obtained from PartitionFinder [103] was used in RAxML-NG v1.2.1 [105] software to the build ML tree. The model was GTR + I + G, which was run 1000 times in total, and the bootstrap value of the ML tree was 100. Figtree v1.4.4 [106] was used to visualize the structure of a tree. Genus names, and GenBank accession numbers and their references for the species used to construct the phylogenetic tree, are found in Table S8.

### 4.5. RNA Extraction and cDNA Synthesis

Four samples each of the 4 °C groups and the 25 °C groups of the four species were used for RNA extraction. RNA was obtained by using the RNA extraction kit from Chengdu Fuji Biological Company (Chengdu, China) according to the manufacturer’s instructions. Next, the concentration of the obtained RNA was measured with infinite M200pro enzyme label and the absorbance at 260 nm and 280 nm was measured to keep the A260/280 value greater than 1.7 [107], and the measured OD value was recorded. The RNA reverse transcription mixture was 10 µL in total, including 2 µL PrimeScript^TM^ RT Master Mix and 8 µL of remaining RNase-Free ddH_2_O containing the RNA sample. The formula: RNA concentration = 500 ng/OD value was used to calculate the required RNA concentration and water amount. Reverse transcription sampling was performed on a super-clean bench, and the sampling process was carried out on ice. After mixing, the PCR reaction was performed using reverse transcription; PCR parameters were 37 °C 15 min, 85 °C 5 s, 4 °C +∞. The obtained cDNA was stored in an ultra-low-temperature freezer at −80 °C.

### 4.6. Quantitative Primer Design and Relative mRNA Quantification

Based on the mitochondrial whole genome sequence of multiple species of Hylidae, as determined by conventional PCR, the gene sequences and lengths of the 13 protein-coding genes were obtained. Primer Primier 6.0 (Primer Biosoft International) was used to design primers based on the complete sequence of *D. japonicus*, *D. immaculata*, *H. annectans*, *H. chinensis* and *H. zhaopingensis*. Appropriate primers were selected for subsequent formal quantitative experiments. The specific primers used in this study are shown in Table S9. Using EASY Dilution, the cDNA from each sample was diluted to 5 different concentrations of 10^−1^, 10^−2^, 10^−3^, 10^−4^ and 10^−5^. Each sample used was 20 µL and included 10 µL SYBR Premix Ex Taq II (2×), 0.4 µL ROX Reference Dye (50×), 0.8 µL forward and reverse primers (10 µMol), 6 µL ddH_2_O and 2 µL RT reactants (cDNA) for RT-qPCR. Quantitative primers were screened in the StepOnePlus™ PCR reaction system under the following conditions: (a) first stage predenaturing at 95 °C, 30 s, one cycle; (b) PCR reaction: 95 °C, 5 s, and 55 °C, 30 s, for 40 cycles; and finally, the formation solution curve was 95 °C, 15 s, 60 °C, 1 min, and 95 °C, 15 s. Real-time fluorescence quantitative PCR analyses using StepOnePlus™ [108], with β-actin used as an internal reference gene, established a standard curve and three technical replicates were performed for each gene. The upstream primer used for β-actin was GATCTGGCATCACACTTTCT, and the downstream was GTGACACCATCACCAGA. cDNA was diluted with double-distilled water (ddH_2_O), and the dilution ratio was based on the efficiency of primer amplification. In this study, cDNA was diluted tenfold. Quantitative experiments were performed on a super-clean bench using the SYBR Premix ExTaq kit. The system used was the same as the primer screening system. The design reaction conditions were as follows: 95 °C 30 s, (95 °C 5 s, 55 °C 30 s) for 40 cycles, then 95 °C 15 s, 60 °C 1 min, 95 °C 15 s.

### 4.7. Data Analysis

Transcript levels of the 13 mitochondrial protein-coding genes were measured using RT-qPCR and StepOne Software v2.1 [108] software, with β-actin as the reference gene, and the expression of each gene was calculated as 2^−ΔΔCt^. The values for each group were saved as mean ± SE, and SPSS21.0 (SPSS, Inc., Chicago, IL, USA) was used to analyze differences between the values by an independent sample *t*-test, where *p* < 0.05 was accepted as a significance difference for *n* = 3 repeats [109,110]. We calculated the multiple relationships between the relative expression levels of the low-temperature groups and the control groups. The expression levels of the 13 mitochondrial protein-coding genes obtained were then mapped with Origin2021 (Origin Lab, Northampton, MA, USA) to compare the changes in gene expression levels.

## 5. Conclusions

It may be a long evolutionary process for amphibians to adapt to lower temperatures. Different species of the Hylidae have different response modes when subjected to low-temperature stress. *D. immaculata*, *H. annectans* and *H. chinensis*, which are located at slightly higher latitudes, can all enter a state of hypometabolism in response to cold stress, thereby reducing energy consumption and reducing the expression of some genes in mitochondria. However, *H. zhaopingensis*, which lives at low latitudes and has a narrow distribution, appears to have insufficient defense mechanisms against low-temperature damage, which can result in metabolic disorder. In the context of extreme low temperatures occurring randomly in a warm winter, *H. zhaopingensis* is probably the most likely to become locally extinct among the four species. By studying and comparing their expression levels, we can infer which one of these four frogs is more vulnerable to extinction and their adaptive plasticity to the environment.

## Figures and Tables

**Table 1:** The mitogenome composition of the five species.

	Full Length * (bp)	A (%)	T (%)	C (%)	G (%)	A + T (%)	G + C (%)	AT Skew	GC Skew
*Dryophytes japonicus*	17,221	30.2	30.2	25.2	14.4	60.4	39.6	−0.001	−0.273
*Dryophytes immaculata*	18,186	29.4	28.5	27.3	14.9	57.9	42.2	0.015	−0.295
*Hyla annectans*	17,060	30.3	30.5	24.8	14.5	60.8	39.3	−0.004	−0.263
*Hyla chinensis*	17,087	30.2	30	25.4	14.4	60.2	39.8	0.004	−0.276
*Hyla zhaopingensis*	15,812	29.8	32.2	23.9	14.1	62	38	−0.038	−0.257

Annotation: * means the whole genome except partial CR.

**Table 2:** Genetic distances of six species of Hylidae.

	KY700829 *Dryophytes suweonensis*	KY419887 *Dryophytes suweonensis*	KX854020 *Dryophytes suweonensis*	*Dryophytes * *japonicus*	*Dryophytes * *immaculata*	AB303949.1 *Dryophytes * *japonicus*
KY700829 *Dryophytes suweonensis*						
KY419887 *Dryophytes suweonensis*	0.12158					
KX854020 *Dryophytes suweonensis*	0.12183	0.00176				
*Dryophytes japonicus*	0.01276	0.12164	0.12189			
*Dryophytes immaculata*	0.12175	0.00831	0.00812	0.12189		
AB303949.1 *Dryophytes japonicus*	0.02526	0.12061	0.12086	0.02479	0.12046	

## Data Availability

Data to support this study are available from the National Center for Biotechnology Information (https://www.ncbi.nlm.nih.gov) (accessed on 20 June 2023). The GenBank numbers are OR398488-OR398492.

## Supplementary Materials

The following supporting information can be downloaded at: https://www.mdpi.com/article/10.3390/ijms25115967/s1. References [111,112,113,114,115,116] are cited in the Supplementary Materials.
