# Supplementary material for: Differential Mitochondrial Genome Expression of Four Hylid Frog Species under Low-Temperature Stress and Its Relationship with Amphibian Temperature Adaptation
Source: Int J Mol Sci. 2024 May 29;25(11):5967. doi: 10.3390/ijms25115967 (PMC11172996; doi:10.3390/ijms25115967)
Supplement: Supplementary file 1 [file ijms-25-05967-s001.zip › Table S1 Locations of features.pdf]

Table S1 (A) Locations of features in the mtDNA of *Dryophytes japonicus*.

Gene/region	Position	Length （bp）	Spacer（+） overlap（-）	Start codon	Stop codon	Strand
trnL1	1-72	72	0			H
trnT	73-141	69	0			H
trnP	141-209	69	-1			L
trnF	209-276	68	-1			H
12S rRNA	277-1214	938	0			H
trnV	1210-1278	69	-5			H
16S rRNA	1279-2878	1600	0			H
trnL2	2879-2951	73	0			H
ND1	2952-3912	961	0	TTG	T	H
trnI	3913-3983	71	0			H
trnQ	3983-4053	71	-1			L
trnM	4053-4121	69	-1			H
ND2	4122-5156	1035	0	ATT	AGA	H
trnW	5160-5229	70	+3			H
trnA	5230-5298	69	0			L
trnN	5299-5371	73	0			L
O_L	5374-5399	26	+2			
trnC	5399-5462	64	-1			L
trnY	5463-5532	70	0			L
COX1	5537-7078	1542	+4	ATA	AGG	H
trnS2	7080-7150	71	+1			L
trnD	7152-7220	69	+1			H
COX2	7222-7909	688	+1	ATG	T	H
trnK	7910-7981	72	0			H
ATP8	7982-8146	165	0	ATG	TAA	H
ATP6	8137-8820	684	-10	ATG	TAA	H
COX3	8820-9604	785	-1	ATG	TA	H
trnG	9605-9673	69	0			H
ND3	9674-10013	340	0	ATG	T	H
trnR	10014-10081	68	0			H
ND4L	10082-10384	303	0	ATG	TAA	H
ND4	10378-11742	1365	-7	ATG	TAG	H
trnH	11743-11811	69	0			H
trnS1	11812-11878	67	0			H
ND5	11914-13716	1789	+35	ATG	AGA	H
ND6	13700-14197	498	-17	ATG	AGA	L
trnE	14198-14265	68	0			L
Cytb	14268-15416	1149	+2	ATG	TAG	H
CR	15417-17221	1805	0			

Table S1 (B) Locations of features in the mtDNA of *Dryophytes immaculata*.

Gene/region	Position	Length （bp）	Spacer（+） overlap（-）	Start codon	Stop codon	Strand
CR1	1-940	940	0			
trnL1	941-1012	72	0			H
trnT	1013-1081	69	0			H
trnP	1081-1149	69	-1			L
trnF	1149-1216	68	-1			H
12S rRNA	1217-2152	936	0			H
trnV	2148-2216	69	-5			H
16S rRNA	2217-3817	1601	0			H
trnL2	3818-3890	73	0			H
ND1	3891-4851	961	0	TTG	T	H
trnI	4852-4922	71	0			H
trnQ	4922-4992	71	-1			L
trnM	4992-5060	69	-1			H
ND2	5061-6095	1035	0	ATT	AGA	H
trnW	6104-6173	70	+8			H
trnA	6174-6242	69	0			L
trnN	6243-6315	73	0			L
O_L	6318-6343	26	+2			
trnC	6343-6406	64	-1			L
trnY	6407-6476	70	0			L
COX1	6481-8022	1542	+4	ATA	AGA	H
trnS2	8024-8094	71	+1			L
trnD	8096-8164	69	+1			H
COX2	8166-8853	688	+1	ATG	T	H
trnK	8854-8925	72	0			H
ATP8	8926-9090	165	0	ATG	TAA	H
ATP6	9081-9764	684	-10	ATG	TAA	H
COX3	9764-10548	785	-1	ATG	TA	H
trnG	10549-10617	69	-1			H
ND3	10621-10957	340	0	ATT	T	H
trnR	10958-11025	68	0			H
ND4L	11026-11328	303	0	ATG	TAA	H
ND4	11322-12686	1365	-7	ATG	TAA	H
trnH	12687-12755	69	0			H
trnS1	12756-12822	67	0			H
ND5	12858-14660	1789	+35	ATG	AGA	H
ND6	14644-15141	498	-17	ATG	AGA	L
trnE	15142-15209	68	0			L
Cytb	15212-16360	1149	+2	ATG	TAG	H
CR2	16361-18186	1826	0			

Table S1 (C) Locations of features in the mtDNA of *Hyla annectans*.

Gene/region	Position	Length （bp）	Spacer （+） overlap （-）	Start codon	Stop codon	Strand
trnL1	1-72	72	0			H
trnT	73-143	71	0			H
trnP	143-211	69	-1			L
trnF	211-278	68	-1			H
12S rRNA	279-1211	933	0			H
trnV	1212-1280	69	0			H
16S rRNA	1281-2876	1596	0			H
trnL2	2877-2949	73	0			H
ND1	2950-3910	961	0	TTG	T	H
trnI	3911-3981	71	0			H
trnQ	3981-4051	71	-1			L
trnM	4051-4119	69	-1			H
ND2	4120-5154	1035	0	ATT	AGA	H
trnW	5161-5230	70	+6			H
trnA	5231-5299	69	0			L
trnN	5300-5372	73	0			L
O_L	5375-5398	24	+2			
trnC	5398-5461	64	-1			L
trnY	5462-5531	70	0			L
COX1	5536-7077	1542	+4	ATA	AGA	H
trnS2	7079-7149	71	+1			L
trnD	7151-7219	69	+1			H
COX2	7221-7908	688	+1	ATG	T	H
trnK	7909-7980	72	0			H
ATP8	7981-8145	165	0	ATG	TAA	H
ATP6	8121-8819	699	-25	ATC	TAA	H
COX3	8819-9603	785	-1	ATG	TA	H
trnG	9604-9672	69	0			H
ND3	9673-10012	340	0	ATG	T	H
trnR	10013-10081	69	0			H
ND4L	10084-10386	303	+2	ATG	TAG	H
ND4	10380-11744	1365	-7	ATG	TAA	H
trnH	11745-11813	69	0			H
trnS1	11814-11880	67	0			H
ND5	11917-13719	1803	+36	ATG	AGA	H
ND6	13703-14200	498	-17	ATG	AGA	L
trnE	14201-14268	68	0			L
Cytb	14271-15419	1149	+2	ATG	TAG	H
CR	15420-17060	1641	0			

Table S1 (D) Locations of features in the mtDNA of *Hyla chinensis*.

Gene/region	Position	Length （bp）	Spacer （+） overlap （-）	Start codon	Stop codon	Strand
CR1	1-118	118	0			
trnL1	119-190	72	0			H
trnT	191-261	71	0			H
trnP	261-329	69	-1			L
trnF	329-396	68	-1			H
12S rRNA	397-1334	938	0			H
trnV	1330-1398	69	-5			H
16S rRNA	1399-2993	1595	0			H
trnL2	2994-3066	73	0			H
ND1	3067-4027	961	0	TTG	T	H
trnI	4028-4098	71	0			H
trnQ	4098-4168	71	-1			L
trnM	4168-4236	69	-1			H
ND2	4237-5271	1035	0	ATT	AGA	H
trnW	5277-5346	70	+5			H
trnA	5347-5415	69	0			L
trnN	5416-5488	73	0			L
O_L	5491-5514	24	+2			
trnC	5514-5577	64	-1			L
trnY	5578-5647	70	0			L
COX1	5652-7193	1542	+4	ATA	AGA	H
trnS2	7195-7265	71	+1			L
trnD	7267-7335	69	+1			H
COX2	7337-8024	688	+1	ATG	T	H
trnK	8025-8096	72	0			H
ATP8	8097-8261	165	0	ATG	TAA	H
ATP6	8237-8935	699	-25	ATC	TAA	H
COX3	8935-9719	785	-1	ATG	TA	H
trnG	9720-9788	69	0			H
ND3	9789-10128	340	0	ATG	T	H
trnR	10129-10197	69	0			H
ND4L	10199-10501	303	+1	ATG	TAG	H
ND4	10495-11859	1365	-7	ATG	TAA	H
trnH	11860-11928	69	0			H
trnS1	11929-11995	67	0			H
ND5	12031-13833	1789	+35	ATG	AGA	H
ND6	13817-14314	498	-17	ATG	AGA	L
trnE	14315-14382	68	0			L
Cytb	14385-15533	1149	+2	ATG	TAA	H
CR2	15534-17087	1554	0			

Table S1 (E) Locations of features in the mtDNA of *Hyla zhaopingensis*.

Gene/region	Position	Length （bp）	Spacer （+） overlap （-）	Start codon	Stop codon	Strand
trnL1	1-72	72	0			H
trnT	73-141	69	0			H
trnP	141-209	69	-1			L
trnF	209-276	68	-1			H
12S rRNA	277-1208	932	0			H
trnV	1209-1277	69	0			H
16S rRNA	1278-2877	1600	0			H
trnL2	2878-2950	73	0			H
ND1	2951-3911	961	0	TTG	T	H
trnI	3912-3983	72	0			H
trnQ	3983-4054	72	-1			L
trnM	4054-4122	69	-1			H
ND2	4123-5157	1035	0	ATT	AGA	H
trnW	5164-5233	70	+6			H
trnA	5234-5302	69	0			L
trnN	5303-5375	73	0			L
O_L	5378-5402	25	+2			
trnC	5402-5465	64	-1			L
trnY	5466-5536	71	0			L
COX1	5541-7082	1542	+4	ATA	AGA	H
trnS2	7084-7154	71	+1			L
trnD	7156-7224	69	+1			H
COX2	7226-7913	688	+1	ATG	T	H
trnK	7914-7985	72	0			H
ATP8	7986-8150	165	0	ATG	TAA	H
ATP6	8126-8824	699	-25	ATC	TAA	H
COX3	8824-9608	785	-1	ATG	TA	H
trnG	9609-9673	69	0			H
ND3	9678-10017	340	0	ATG	T	H
trnR	10018-10086	69	0			H
ND4L	10089-10391	303	+2	ATG	TAG	H
ND4	10385-11749	1365	-7	ATG	TAA	H
trnH	11750-11818	69	0			H
trnS1	11819-11885	67	0			H
ND5	11919-13721	1789	+33	ATG	AGA	H
ND6	13705-14202	498	-17	ATG	AGA	L
trnE	14203-14270	68	0			L
Cytb	14273-15421	1149	+2	ATG	TAA	H
CR	15422-15812	391	0			
