# Supplementary material for: Differential Mitochondrial Genome Expression of Four Hylid Frog Species under Low-Temperature Stress and Its Relationship with Amphibian Temperature Adaptation
Source: Int J Mol Sci. 2024 May 29;25(11):5967. doi: 10.3390/ijms25115967 (PMC11172996; doi:10.3390/ijms25115967)
Supplement: Supplementary file 1 [file ijms-25-05967-s001.zip › Table S2 RSCU.pdf]

Table S2 (A). The codon number and relative synonymous codon usage in mitochondrial protein-coding genes of *Dryophytes japonicus*.

Codon	Count	RSCU	Codon	Count	RSCU	Codon	Count	RSCU	Codon	Count	RSCU
UUU(F)	143	1.13	UCU(S)	82	1.58	UAU(Y)	55	0.94	UGU(C)	8	0.48
UUC(F)	109	0.87	UCC(S)	73	1.4	UAC(Y)	62	1.06	UGC(C)	25	1.52
UUA(L)	132	1.34	UCA(S)	96	1.85	UAA (*)	3	1.5	UGA(W)	93	1.68
UUG(L)	35	0.35	UCG(S)	7	0.13	UAG (*)	2	1	UGG(W)	18	0.32
CUU(L)	147	1.49	CCU(P)	45	0.9	CAU(H)	37	0.8	CGU(R)	13	0.74
CUC(L)	87	0.88	CCC(P)	55	1.1	CAC(H)	56	1.2	CGC(R)	10	0.57
CUA(L)	160	1.62	CCA(P)	90	1.8	CAA(Q)	84	1.91	CGA(R)	43	2.46
CUG(L)	31	0.31	CCG(P)	10	0.2	CAG(Q)	4	0.09	CGG(R)	4	0.23
AUU(I)	227	1.32	ACU(T)	69	1.04	AAU(N)	68	1.05	AGU(S)	17	0.33
AUC(I)	118	0.68	ACC(T)	83	1.25	AAC(N)	62	0.95	AGC(S)	37	0.71
AUA(M)	127	1.51	ACA(T)	107	1.61	AAA(K)	79	1.78	AGA (*)	2	1
AUG(M)	41	0.49	ACG(T)	7	0.11	AAG(K)	10	0.22	AGG (*)	1	0.5
GUU(V)	62	1.31	GCU(A)	94	1.21	GAU(D)	33	0.92	GGU(G)	47	0.85
GUC(V)	48	1.01	GCC(A)	112	1.44	GAC(D)	39	1.08	GGC(G)	48	0.86
GUA(V)	58	1.22	GCA(A)	101	1.29	GAA(E)	73	1.55	GGA(G)	84	1.51
GUG(V)	22	0.46	GCG(A)	5	0.06	GAG(E)	21	0.45	GGG(G)	43	0.77

Table S2 (B). The codon number and relative synonymous codon usage in mitochondrial protein-coding genes of *Dryophytes immaculata*.

Codon	Count	RSCU	Codon	Count	RSCU	Codon	Count	RSCU	Codon	Count	RSCU
UUU(F)	121	0.96	UCU(S)	72	1.38	UAU(Y)	38	0.67	UGU(C)	12	0.73
UUC(F)	130	1.04	UCC(S)	81	1.55	UAC(Y)	76	1.33	UGC(C)	21	1.27
UUA(L)	116	1.16	UCA(S)	102	1.96	UAA (*)	4	2	UGA(W)	90	1.62
UUG(L)	32	0.32	UCG(S)	8	0.15	UAG (*)	1	0.5	UGG(W)	21	0.38
CUU(L)	125	1.25	CCU(P)	37	0.74	CAU(H)	31	0.67	CGU(R)	11	0.63
CUC(L)	110	1.1	CCC(P)	84	1.69	CAC(H)	62	1.33	CGC(R)	10	0.57
CUA(L)	158	1.58	CCA(P)	65	1.31	CAA(Q)	78	1.75	CGA(R)	43	2.46
CUG(L)	60	0.6	CCG(P)	13	0.26	CAG(Q)	11	0.25	CGG(R)	6	0.34
AUU(I)	183	1.07	ACU(T)	61	0.91	AAU(N)	54	0.82	AGU(S)	11	0.21
AUC(I)	159	0.93	ACC(T)	103	1.54	AAC(N)	78	1.18	AGC(S)	39	0.75
AUA(M)	118	1.52	ACA(T)	100	1.49	AAA(K)	75	1.7	AGA (*)	3	1.5
AUG(M)	37	0.48	ACG(T)	4	0.06	AAG(K)	13	0.3	AGG (*)	0	0
GUU(V)	62	1.27	GCU(A)	77	0.98	GAU(D)	25	0.68	GGU(G)	35	0.62
GUC(V)	56	1.15	GCC(A)	137	1.75	GAC(D)	48	1.32	GGC(G)	54	0.96
GUA(V)	50	1.03	GCA(A)	90	1.15	GAA(E)	70	1.49	GGA(G)	84	1.5
GUG(V)	27	0.55	GCG(A)	9	0.12	GAG(E)	24	0.51	GGG(G)	51	0.91

Table S2 (C). The codon number and relative synonymous codon usage in mitochondrial protein-coding genes of *Hyla annectans*.

Codon	Count	RSCU	Codon	Count	RSCU	Codon	Count	RSCU	Codon	Count	RSCU
UUU(F)	169	1.36	UCU(S)	81	1.57	UAU(Y)	64	1.08	UGU(C)	11	0.71
UUC(F)	80	0.64	UCC(S)	70	1.35	UAC(Y)	54	0.92	UGC(C)	20	1.29
UUA(L)	185	1.81	UCA(S)	98	1.9	UAA (*)	3	1.5	UGA(W)	100	1.8
UUG(L)	35	0.34	UCG(S)	10	0.19	UAG (*)	2	1	UGG(W)	11	0.2
CUU(L)	114	1.12	CCU(P)	44	0.92	CAU(H)	36	0.78	CGU(R)	6	0.34
CUC(L)	86	0.84	CCC(P)	65	1.35	CAC(H)	56	1.22	CGC(R)	9	0.51
CUA(L)	157	1.54	CCA(P)	70	1.46	CAA(Q)	78	1.71	CGA(R)	50	2.86
CUG(L)	35	0.34	CCG(P)	13	0.27	CAG(Q)	13	0.29	CGG(R)	5	0.29
AUU(I)	216	1.28	ACU(T)	75	1.11	AAU(N)	66	0.99	AGU(S)	22	0.43
AUC(I)	121	0.72	ACC(T)	80	1.18	AAC(N)	68	1.01	AGC(S)	29	0.56
AUA(M)	116	1.46	ACA(T)	109	1.61	AAA(K)	76	1.73	AGA (*)	3	1.5
AUG(M)	43	0.54	ACG(T)	7	0.1	AAG(K)	12	0.27	AGG (*)	0	0
GUU(V)	62	1.33	GCU(A)	83	1.04	GAU(D)	28	0.76	GGU(G)	39	0.7
GUC(V)	46	0.99	GCC(A)	116	1.45	GAC(D)	46	1.24	GGC(G)	49	0.88
GUA(V)	66	1.42	GCA(A)	115	1.44	GAA(E)	72	1.58	GGA(G)	89	1.6
GUG(V)	12	0.26	GCG(A)	6	0.07	GAG(E)	19	0.42	GGG(G)	45	0.81

Table S2 (D). The codon number and relative synonymous codon usage in mitochondrial protein-coding genes of *Hyla chinensis*.

Codon	Count	RSCU	Codon	Count	RSCU	Codon	Count	RSCU	Codon	Count	RSCU
UUU(F)	155	1.25	UCU(S)	84	1.64	UAU(Y)	66	1.16	UGU(C)	15	0.91
UUC(F)	93	0.75	UCC(S)	64	1.25	UAC(Y)	48	0.84	UGC(C)	18	1.09
UUA(L)	166	1.65	UCA(S)	104	2.03	UAA (*)	4	2	UGA(W)	92	1.64
UUG(L)	33	0.33	UCG(S)	6	0.12	UAG (*)	1	0.5	UGG(W)	20	0.36
CUU(L)	116	1.15	CCU(P)	43	0.88	CAU(H)	34	0.7	CGU(R)	8	0.46
CUC(L)	95	0.95	CCC(P)	65	1.33	CAC(H)	63	1.3	CGC(R)	8	0.46
CUA(L)	157	1.56	CCA(P)	81	1.65	CAA(Q)	79	1.76	CGA(R)	49	2.84
CUG(L)	36	0.36	CCG(P)	7	0.14	CAG(Q)	11	0.24	CGG(R)	4	0.23
AUU(I)	213	1.26	ACU(T)	73	1.05	AAU(N)	67	0.98	AGU(S)	17	0.33
AUC(I)	126	0.74	ACC(T)	95	1.37	AAC(N)	70	1.02	AGC(S)	32	0.63
AUA(M)	135	1.64	ACA(T)	99	1.43	AAA(K)	77	1.77	AGA (*)	3	1.5
AUG(M)	30	0.36	ACG(T)	10	0.14	AAG(K)	10	0.23	AGG (*)	0	0
GUU(V)	52	1.17	GCU(A)	76	0.95	GAU(D)	32	0.86	GGU(G)	32	0.58
GUC(V)	42	0.94	GCC(A)	132	1.66	GAC(D)	42	1.14	GGC(G)	66	1.19
GUA(V)	64	1.44	GCA(A)	104	1.3	GAA(E)	71	1.54	GGA(G)	76	1.38
GUG(V)	20	0.45	GCG(A)	7	0.09	GAG(E)	21	0.46	GGG(G)	47	0.85

Table S2 (E). The codon number and relative synonymous codon usage in mitochondrial protein-coding genes of *Hyla zhaopingensis*.

Codon	Count	RSCU	Codon	Count	RSCU	Codon	Count	RSCU	Codon	Count	RSCU
UUU(F)	183	1.4	UCU(S)	93	1.85	UAU(Y)	73	1.23	UGU(C)	13	0.84
UUC(F)	79	0.6	UCC(S)	57	1.13	UAC(Y)	46	0.77	UGC(C)	18	1.16
UUA(L)	204	2.04	UCA(S)	95	1.89	UAA (*)	5	2.22	UGA(W)	102	1.82
UUG(L)	28	0.28	UCG(S)	7	0.14	UAG (*)	1	0.44	UGG(W)	10	0.18
CUU(L)	142	1.42	CCU(P)	72	1.48	CAU(H)	38	0.84	CGU(R)	14	0.81
CUC(L)	67	0.67	CCC(P)	39	0.8	CAC(H)	52	1.16	CGC(R)	8	0.46
CUA(L)	141	1.41	CCA(P)	78	1.6	CAA(Q)	79	1.74	CGA(R)	42	2.43
CUG(L)	19	0.19	CCG(P)	6	0.12	CAG(Q)	12	0.26	CGG(R)	5	0.29
AUU(I)	255	1.53	ACU(T)	95	1.37	AAU(N)	68	1.01	AGU(S)	25	0.5
AUC(I)	79	0.47	ACC(T)	79	1.14	AAC(N)	67	0.99	AGC(S)	25	0.5
AUA(M)	127	1.57	ACA(T)	94	1.35	AAA(K)	79	1.82	AGA (*)	3	1.33
AUG(M)	35	0.43	ACG(T)	10	0.14	AAG(K)	8	0.18	AGG (*)	0	0
GUU(V)	70	1.48	GCU(A)	101	1.29	GAU(D)	36	0.99	GGU(G)	43	0.76
GUC(V)	44	0.93	GCC(A)	109	1.4	GAC(D)	37	1.01	GGC(G)	58	1.03
GUA(V)	63	1.33	GCA(A)	95	1.22	GAA(E)	76	1.67	GGA(G)	80	1.42
GUG(V)	12	0.25	GCG(A)	7	0.09	GAG(E)	15	0.33	GGG(G)	44	0.78
