# Supplementary material for: Differential Mitochondrial Genome Expression of Four Hylid Frog Species under Low-Temperature Stress and Its Relationship with Amphibian Temperature Adaptation
Source: Int J Mol Sci. 2024 May 29;25(11):5967. doi: 10.3390/ijms25115967 (PMC11172996; doi:10.3390/ijms25115967)
Supplement: Supplementary file 1 [file ijms-25-05967-s001.zip › Table S4 COX1 distance.pdf]

TableS4 (A) Genetic distance table for quantitative *COX1* of *Dryophytes immaculata*

	DLAHCZ3	DLAHCZ4	DLAHCZ5	DLAHCZ7	DLAHCZ11	DLAHCZ12	DLAHCZ13	DLAHCZ14
DLAHCZ3								
DLAHCZ4	0.0071910757							
DLAHCZ5	0.0017913137	0.0053884515						
DLAHCZ7	0.0053884515	0.0090116558	0.0035906797					
DLAHCZ11	0.0017913137	0.0053884515	0.0000000000	0.0035906797				
DLAHCZ12	0.0017921166	0.0090026719	0.0035866478	0.0071967717	0.0035866478			
DLAHCZ13	0.0017921166	0.0090026719	0.0035866478	0.0071967717	0.0035866478	0.0035906797		
DLAHCZ14	0.0035874565	0.0071910757	0.0017913137	0.0053884515	0.0017913137	0.0053860196	0.0053860196	

TableS4 (B) Genetic distance table for quantitative *COX1* of *Hyla annectans*

	DLGZAS1	DLGZAS2	DLGZAS3	DLGZAS6	DLGZAS11	DLGZAS12	DLGZAS13	DLGZAS14
DLGZAS1								
DLGZAS2	0.0044543724							
DLGZAS3	0.0022222259	0.0022222259						
DLGZAS6	0.0022222259	0.0022222259	0.0000000000					
DLGZAS11	0.0044543724	0.0000000000	0.0022222259	0.0022222259				
DLGZAS12	0.0022222259	0.0022222259	0.0000000000	0.0000000000	0.0022222259			
DLGZAS13	0.0066965287	0.0066965287	0.0044543724	0.0044543724	0.0066965287	0.0044543724		
DLGZAS14	0.0022222259	0.0022222259	0.0000000000	0.0000000000	0.0022222259	0.0000000000	0.0044543724	

TableS4 (C) Genetic distance table for quantitative *COX1* of *Hyla chinensis*.

	DLSZWZ2	DLSZWZ3	DLSZWZ4	DLSZWZ5	DLSZWZ11	DLSZWZ13	DLSZWZ14	DLSZWZ15
DLSZWZ2								
DLSZWZ3	0.0054845157							
DLSZWZ4	0.0000000000	0.0054845157						
DLSZWZ5	0.0054845157	0.0000000000	0.0054845157					
DLSZWZ11	0.0036496512	0.0018214956	0.0036496512	0.0018214956				
DLSZWZ13	0.0000000000	0.0054845157	0.0000000000	0.0054845157	0.0036496512			
DLSZWZ14	0.0000000000	0.0054845157	0.0000000000	0.0054845157	0.0036496512	0.0000000000		
DLSZWZ15	0.0000000000	0.0054845157	0.0000000000	0.0054845157	0.0036496512	0.0000000000	0.0000000000	

TableS4 (D) Genetic distance table for quantitative *COX1* of *Hyla zhaopingensis*.

	DLGDMM1	DLGDMM2	DLGDMM3	DLGDMM4	DLGDMM11	DLGDMM13	DLGDMM18	DLGDMM19
DLGDMM1								
DLGDMM2	0.0000000000							
DLGDMM3	0.0026229557	0.0026229557						
DLGDMM4	0.0026229557	0.0026229557	0.0000000000					
DLGDMM11	0.0000000000	0.0000000000	0.0026229557	0.0026229557				
DLGDMM13	0.0000000000	0.0000000000	0.0026229557	0.0026229557	0.0000000000			
DLGDMM18	0.0000000000	0.0000000000	0.0026229557	0.0026229557	0.0000000000	0.0000000000		
DLGDMM19	0.0000000000	0.0000000000	0.0026229557	0.0026229557	0.0000000000	0.0000000000	0.0000000000	
