# Supplementary material for: Differential Mitochondrial Genome Expression of Four Hylid Frog Species under Low-Temperature Stress and Its Relationship with Amphibian Temperature Adaptation
Source: Int J Mol Sci. 2024 May 29;25(11):5967. doi: 10.3390/ijms25115967 (PMC11172996; doi:10.3390/ijms25115967)
Supplement: Supplementary file 1 [file ijms-25-05967-s001.zip › Table S5 The third codon saturation.pdf]

Table S5. The third codon saturation results.

NumOTU	Iss	Iss.cSym	T	DF	P	Iss.cAsym	T	DF	P
4	0.688	0.849	18.655	3503	0.0000	0.836	17.115	3503	0.0000
8	0.678	0.840	19.827	3503	0.0000	0.756	9.516	3503	0.0000
16	0.683	0.826	18.311	3503	0.0000	0.665	2.324	3503	0.0202
32	0.686	0.808	16.081	3503	0.0000	0.551	17.953	3503	0.0000
