# Supplementary material for: Differential Mitochondrial Genome Expression of Four Hylid Frog Species under Low-Temperature Stress and Its Relationship with Amphibian Temperature Adaptation
Source: Int J Mol Sci. 2024 May 29;25(11):5967. doi: 10.3390/ijms25115967 (PMC11172996; doi:10.3390/ijms25115967)
Supplement: Supplementary file 1 [file ijms-25-05967-s001.zip › Table S6.pdf]

Table S6 PartitionFinder program was used to obtain the best partitioning scheme and the best fit model of first, second and third positions of codon. The full names of all abbreviations are as follows: GTR: general time reversible; I: unchanged site proportion; G: Gamma distribution.

**Nucleotide Sequence Alignments**		
**Subset**	**Subset Partitions**	**Best Model**
Partition 1	NAD4L_codon1, NAD5_codon1, NAD4_codon1, ATP8_codon1, ATP6_codon1, NAD2_codon1	GTR+I+G
Partition 2	NAD1_codon2, ATP6_codon2, NAD4L_codon2, NAD4_codon2, ATP8_codon2, NAD3_codon2, NAD2_codon2, NAD5_codon2	TVM+I+G
Partition 3	NAD4_codon3, ATP6_codon3, ATP8_codon3	TRN+I+G
Partition 4	COX1_codon1	SYM+I+G
Partition 5	COX1_codon2, Cytb_codon2, COX3_codon2, COX2_codon2	K81UF+I+G
Partition 6	COX1_codon3	TRN+G
Partition 7	NAD1_codon1, NAD3_codon1, COX3_codon1, COX2_codon1, Cytb_codon1	SYM+I+G
Partition 8	NAD5_codon3, COX3_codon3, COX2_codon3	TRN+I+G
Partition 9	Cytb_codon3	TRN+I+G
Partition 10	NAD3_codon3, NAD2_codon3, NAD4L_codon3, NAD1_codon3	GTR+I+G
Partition 11	NAD6_codon2, NAD6_codon1	K81UF+I+G
Partition 12	NAD6_codon3	TRN+G
