# Supplementary material for: Differential Mitochondrial Genome Expression of Four Hylid Frog Species under Low-Temperature Stress and Its Relationship with Amphibian Temperature Adaptation
Source: Int J Mol Sci. 2024 May 29;25(11):5967. doi: 10.3390/ijms25115967 (PMC11172996; doi:10.3390/ijms25115967)
Supplement: Supplementary file 1 [file ijms-25-05967-s001.zip › Table S7.pdf]

Table S7. PartitionFinder program was used to obtain the best partitioning scheme and the best fit model of first, second positions of codon. The full names of all abbreviations are as follows: GTR: general time reversible; I: unchanged site proportion; G: Gamma distribution.

Nucleotide Sequence Alignments		
**Subset**	**Subset Partitions**	**Best Model**
Partition 1	NAD2_codon1, ATP8_codon1, ATP6_codon1, NAD3_codon1, NAD4L_codon1, NAD5_codon1, NAD4_codon1	GTR+I+G
Partition 2	NAD1_codon2, ATP6_codon2, NAD4_codon2, NAD4L_codon2, NAD5_codon2, ATP8_codon2, NAD3_codon2, NAD2_codon2	TVM+I+G
Partition 3	COX1_codon1, Cytb_codon1, COX2_codon1, NAD1_codon1, COX3_codon1	SYM+I+G
Partition 4	COX1_codon2, Cytb_codon2, COX2_codon2, COX3_codon2	K81UF+I+G
Partition 5	NAD6_codon2, NAD6_codon1	K81UF+I+G
