# Supplementary material for: Differential Mitochondrial Genome Expression of Four Hylid Frog Species under Low-Temperature Stress and Its Relationship with Amphibian Temperature Adaptation
Source: Int J Mol Sci. 2024 May 29;25(11):5967. doi: 10.3390/ijms25115967 (PMC11172996; doi:10.3390/ijms25115967)
Supplement: Supplementary file 1 [file ijms-25-05967-s001.zip › Table S8 Genbank.pdf]

Table S8. Genus names, GenBank accession numbers and their references for the species used to construct the phylogenetic tree.

Subfamily	genus	Species	GenBank	Length（bp）	Reference
Hylinae	*Dryophytes*	*Dryophytes cinereus*	MF198328	15418	[111]
		Dryophytes squirellus	MF198365	15420	[111]
		Dryophytes suweonensis	KY700829	18288	Unpublished
		Dryophytes femoralis	MW002723	15415	[112]
		Dryophytes suweonensis	KX854020	17448	[60]
		Dryophytes suweonensis	KY419887	18611	[61]
		Dryophytes versicolor	MH087467	18800	[54]
		Dryophytes andersonii	MW002721	15423	[112]
		Dryophytes japonica	AB303949	19519	[62]
		Dryophytes japonica	OR398492	17221	This study
		Dryophytes immaculata	OR398491	18186	This study
	Hyla	*Hyla zhaopingensis*	OR398490	15812	This study
		Hyla annectans	OR398488	17060	This study
		Hyla chinensis	OR398489	17087	This study
		Hyla annectans	KM271781	17973	[63]
		Hyla tsinlingensis	KU601448	18035	[64]
		Hyla sanchiangensis	MK388868	15977	[65]
		Hyla sanchiangensis	MK388867	15675	
		Hyla sanchiangensis	MT561180	15664	[66]
		Hyla sanchiangensis	MZ508281	15694	Direct Submission
		Hyla chinensis	AY458593	18180	[67]
	Osteocephalus	*Osteocephalus taurinus*	OP572191	16548	[68]
		Osteocephalus yasuni	OP572190	17442	
		Osteocephalus leprieurii	OP572188	17314	
		Osteocephalus cabrerai	OP572182	16320	
		Osteocephalus mimeticus	OP572181	18073	
		Osteocephalus vilarsi	OP572180	17399	
		Osteocephalus planiceps	OP572179	17907	
		Osteocephalus leoniae	OP572178	16871	
		Osteocephalus oophagus	OP572173	16643	
		Osteocephalus subtilis	OP572171	16522	
		Osteocephalus helenae	OP425862	18091	
		Osteocephalus taurinus	JX564881	14970	[88]
	Boana	*Boana aff. courtoisae*	MW575899	15840	[113]
		Boana aff. lanciformis	MW575918	17150	
		Boana alfaroi	MW575923	17219	
		Boana almendarizae	MW575920	17439	
		Boana boans	MW575903	17021	
		Boana calcarata	MW575909	16251	
		Boana cinerascens	MW575925	17684	
		Boana dentei	MW575906	17587	
		Boana diabolica	MW575901	14606	
		Boana fasciata	MW575919	17438	
		Boana leucocheila	MW575912	18042	
		Boana maculateralis	MW575922	16301	
		Boana ornatissima	MW575902	17656	
		Boana punctata	MW575907	17899	
		Boana raniceps	MW575911	17180	
		Boana steinbachi	MW575910	18107	
		Boana tetete	MW575921	16107	
		Boana xerophylla	MW575900	18062	
	Dryaderces	*Dryaderces inframaculata*	OP572169	17479	[68]

	Trachycephalus	*Trachycephalus coriaceus*	OP572192	17918	
	Tepuihyla	*Tepuihyla edelcae*	OP572176	21016	
	Pseudis	*Pseudis tocantins*	MH571152	15564	[114]
	Aplastodiscus	*Aplastodiscus* sp.	MW575915	17773	[113]
	Hyloscirtus	*Hyloscirtus* sp.	MW575908	16687	
	Bokermannohyla	*Bokermannohyla alvarengai*	KY829114	17325	[115]
		Bokermannohyla sp.	MW575914	16355	[113]
Phyllomedusinae	*Pithecopus*	*Pithecopus megacephalus*	MG772558	18050	[116]
	Phyllomedusa	*Phyllomedusa tomopyerna*	JX564887	14926	[88]
		Phyllomedusa bahiana	OM460708	16239	Unpublished
Pelodryadinae	*Nyctimystes*	*Nyctimystes kubori*	JX564879	14983	[88]
Outgroups	*Dendrobates*	*Dendrobates auratus*	JX564862	14863	
	Mannophryne	*Mannophryne trinitatis*	JX564878	14939	
	Odontophrynus	*Odontophrynus occidentalis*	JX564880	14908	
	Rhinoderma	*Rhinoderma darwinii*	JX564891	14943	
