# Supplementary material for: Differential Mitochondrial Genome Expression of Four Hylid Frog Species under Low-Temperature Stress and Its Relationship with Amphibian Temperature Adaptation
Source: Int J Mol Sci. 2024 May 29;25(11):5967. doi: 10.3390/ijms25115967 (PMC11172996; doi:10.3390/ijms25115967)
Supplement: Supplementary file 1 [file ijms-25-05967-s001.zip › Table S9 Primers used in this study for 13 protein-coding genes and actin.pdf]

Table S9 (A) Primers used in this study for 13 protein-coding genes and actin in *Dryophytes immaculata*.

Gene name	Forward primers	Reverse primers
Actin	GTGCGTGACATCAAGGAGAAG	AAGAAGGATGGCTGGAAGAGG
ND1	ATCTCCTATGAGGTCACGCTAG	TCGGATTCTCCTTCGGTCAG
ND2	CGACTCTCCTAAGCCTATTCTT	GTTGGTGTGACTGGTAGAAGG
COX1	GCACCTGATATAGCCTTCC	GTAGACGGTTCATCCTGTT
COX2	TCCAACACCAACACCATC	AAGTCGGAGTATTCGTAGC
ATP8	GACTTATCTTCCTTGCATTCTC	GCCAGGATCAGGATTTGTT
ATP6	GCTAACCGTGTCGTAACCATT	ACCGAGAAGGAAGACCATAAGA
COX3	CCTTAATACTGCCGTGCTTCTT	GTAGATGCCGTCAGCGATTG
ND3	CGCCCTTATCCTATCGTTGGT	TAACCGTGCAGAACCGAGTG
ND4L	CGAGCCCATCTTCTGTCA	GCGGAGAGCGTGAGTATA
ND4	GCATAATGGCGTGGTGAG	AGGAAGGAGTCGTTAATAATGG
ND5	GCGTCTTCCTACTAATCCGAAT	CCGAGTTGGCTTGATGTTGA
ND6	CTCTAGTTGCTGAGCCTTATCC	CCCGAACCAATCTCCCACATAT
Cytb	CATCCAGCCAGCGAACTA	TGGTTGTCCTCCGATTCAG

Table S9 (B) Primers used in this study for 13 protein-coding genes and actin in *Hyla annectans*.

Gene name	Forward primers	Reverse primers
Actin	TCTGCTATGTAGCTCTGGACTT	CCTCTGGACACCTGAACCTT
ND1	GAAGTCACACTAGCCTTAATCC	TCATATTATGGCGAGAGGTCAT
ND2	TAATCCTTTCGCCCTCTA	CTGATGCTGCTGCTTGTG
COX1	CCTACTTCACCTCTGCTACCA	AGTATGGCGGCGTCTCATT
COX2	AATTCCGCCTCTTAGAAGTTGA	TTCTGAGCATTGTCCGTAGTAA
ATP8	TCCTTGCATTCTCACCAATT	TAAGCTCAAGGTCAGGATCAA
ATP6	GCAATCGCTGACTAACTAAC	GGTGAATGTGTAAGGAAGGA
COX3	AGCCTGGCACCCTCCTAT	AATGCGGAGATTCCTGTT
ND3	CAGCACGCCTTCCTTATT	GCGTGGGAAGCAACAAAG
ND4L	TTCACTCCACCGAGCACAC	TTCGCAGGCAGAAAGGGTTA
ND4	TGTCTCGCAAACACCTCA	GCTGCGGATAGTGGAAGA
ND5	CCCCTACTCTTAGCCCTCA	CGGATTAGGAGAAATACGC
ND6	TAGTAGCTGAGCCTTATCCTGA	CCTCCGACTAATCACCATAACC
Cytb	AATAACGGCTGACTCTTACGAA	GGCTGTTGCTATGACTAGGAAT

Table S9 (C) Primers used in this study for 13 protein-coding genes and actin in *Hyla chinensis*.

Gene name	Forward primers	Reverse primers
Actin	GTGCGTGACATCAAGGAGAAG	AAGAAGGATGGCTGGAAGAGG
ND1	CGATTGCTGACGGTGTTAAGT	ATGTCACAGAGTGGAGTAGGC
ND2	ACCACTGAATCTTAGCCTGAAT	TGCTGCTGCTTGTGTTAGAA
COX1	ATGTCATCGTCACTGCTCAC	CTGCTGAGGCTAAGAGAAGAAG
COX2	TCCGCCTCCTAGAAGTTGATAA	ATCTCTGAGCACTGTCCATAGT
ATP8	CTTCCTCGTATTCTCACCAACT	TCAAGGTCAGGGTCAGGATTT
ATP6	GTAACCAACTCACTGCCTCC	GTGTCCAGCGGTCAGATTAG
COX3	TTCGTGGCAACAGGATTCC	GTATCATGCGGCTGCTTCA
ND3	CTTCTTCTTCCCACGCCATG	TGCTCATTCTAATCCTCCTTGG
ND4L	CTCTAACCCACGAATCCTGACT	GGCTCATAGACCTAAGGCTACA
ND4	CTAAGCGGTTGTGGGAAGTTAT	CTGTCAATCAGCAAGTGAGGAT
ND5	GCCTGAGCATTAGCACTAACT	TCATTGATGGAGACGATTGAGT
ND6	TGATGCTTATGTTGGCGATTGA	CCTCTAGCACAGCGAATAACG
Cytb	CGGCGTAATCCTACTGTTCCT	GTGGCGTTGTCTACTGAGAATC

Table S9 (D) Primers used in this study for 13 protein-coding genes and actin in *Hyla zhaopingensis*.

Gene name	Forward primers	Reverse primers
Actin	TCTGCTATGTAGCTCTGGACTT	CCTCTGGACACCTGAACCTT
ND1	CGACCATCAAACTCATCTCAAA	GTTAGAGGCTCATCCTGACC
ND2	GCAGCAGCATCAGCCTTAA	TGTCAGTCCTTGTAGAACTTCA
COX1	GGCATACCACGTCGATATTCTG	CTGCGGAGGAGAGGATTCATT
COX2	TCGTGAGCAGTGCCATCT	ATCTCTGAACATTGACCGTAGT
ATP8	ATGCCACAATTAGACCC	GTCAGGATCAGGATTTACTT
ATP6	GCCACAAATGAGCCCTAATTCT	GTGTCCAAGAGATGCTGTAAGT
COX3	GGACATCGTTCGTGAAGGAA	TGTTGGTGGTCAGCATTCTC
ND3	GGTCAGCACGACTTCCATATTC	GCTCAAGGTGTAGGTAGAAGGA
ND4L	CTCTTGTCAGCTCTCTTGTGTT	AGGTTATCCGTGCCGTGAG
ND4	CTTAGTCATCGCAGCATCACTT	AGTCATCAAGCCGCAGATAGT
ND5	TTGTGTCTCCGTAGGCAGTT	GGCTGTAAATGAGGTGGCAAT
ND6	GCGTACTCAGCGGCATTAG	GCCTCTGATCCACCTCCAAT
Cytb	ATCTTCACATTGGGCGAGGA	GCAGCGGATAGGAGGTTAGTA
