# Supplementary figures and images for: Differential Mitochondrial Genome Expression of Four Hylid Frog Species under Low-Temperature Stress and Its Relationship with Amphibian Temperature Adaptation
Source: Int J Mol Sci. 2024 May 29;25(11):5967. doi: 10.3390/ijms25115967 (PMC11172996; doi:10.3390/ijms25115967)

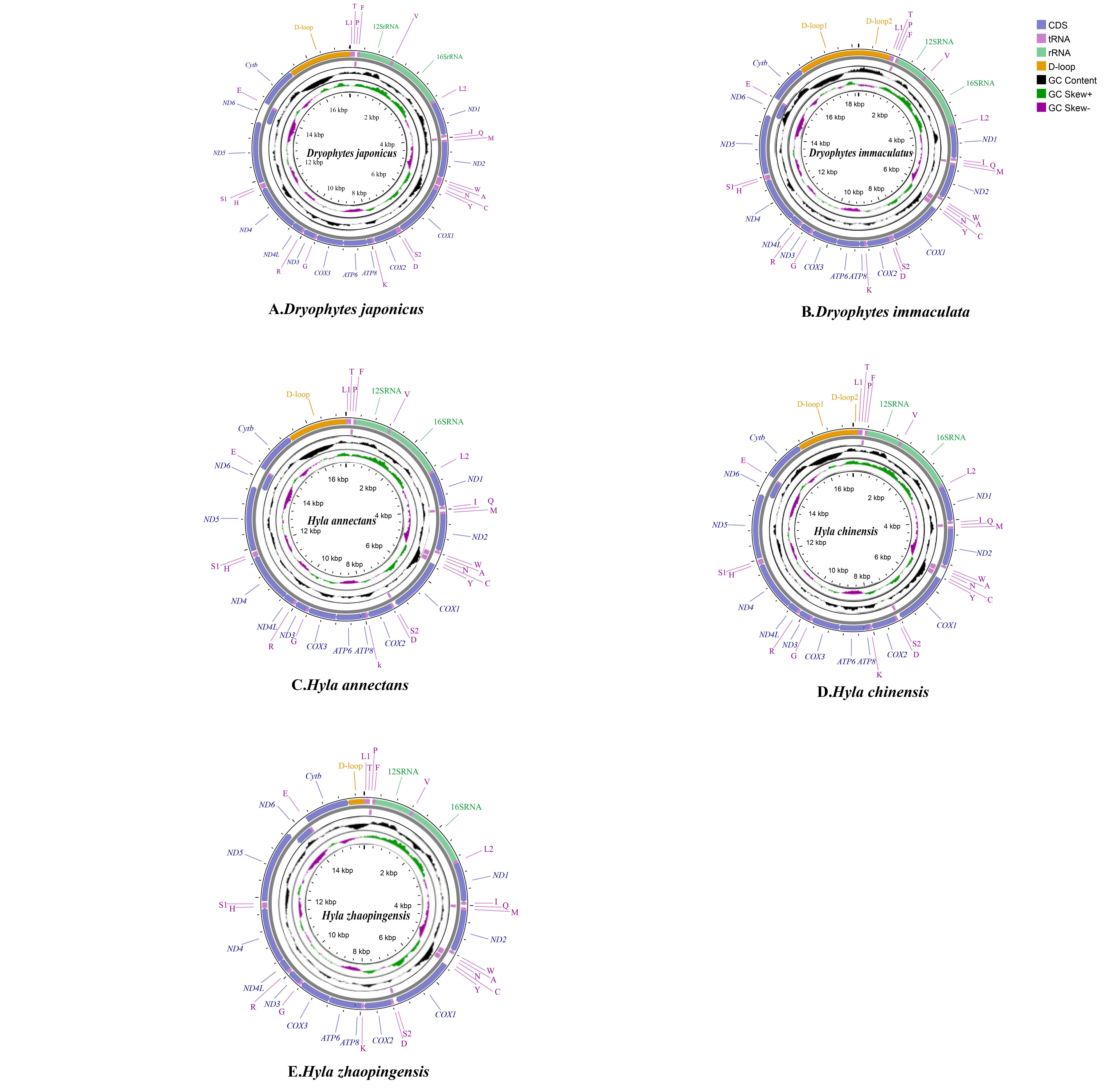

Supplement: Supplementary file 1 [file ijms-25-05967-s001.zip › Figure S1 CGview.png]

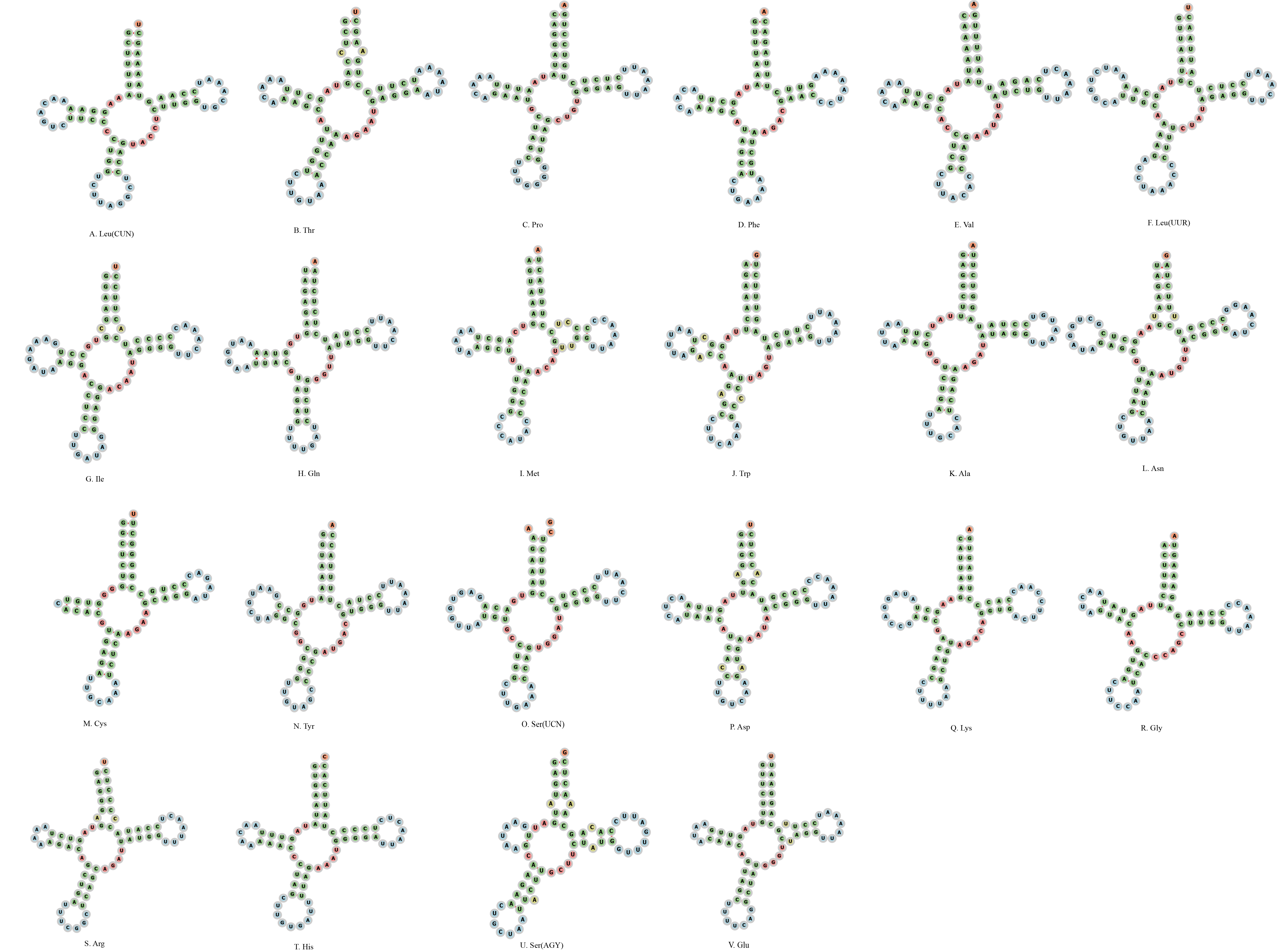

Supplement: Supplementary file 1 [file ijms-25-05967-s001.zip › Figure S2 AHCZ.png]

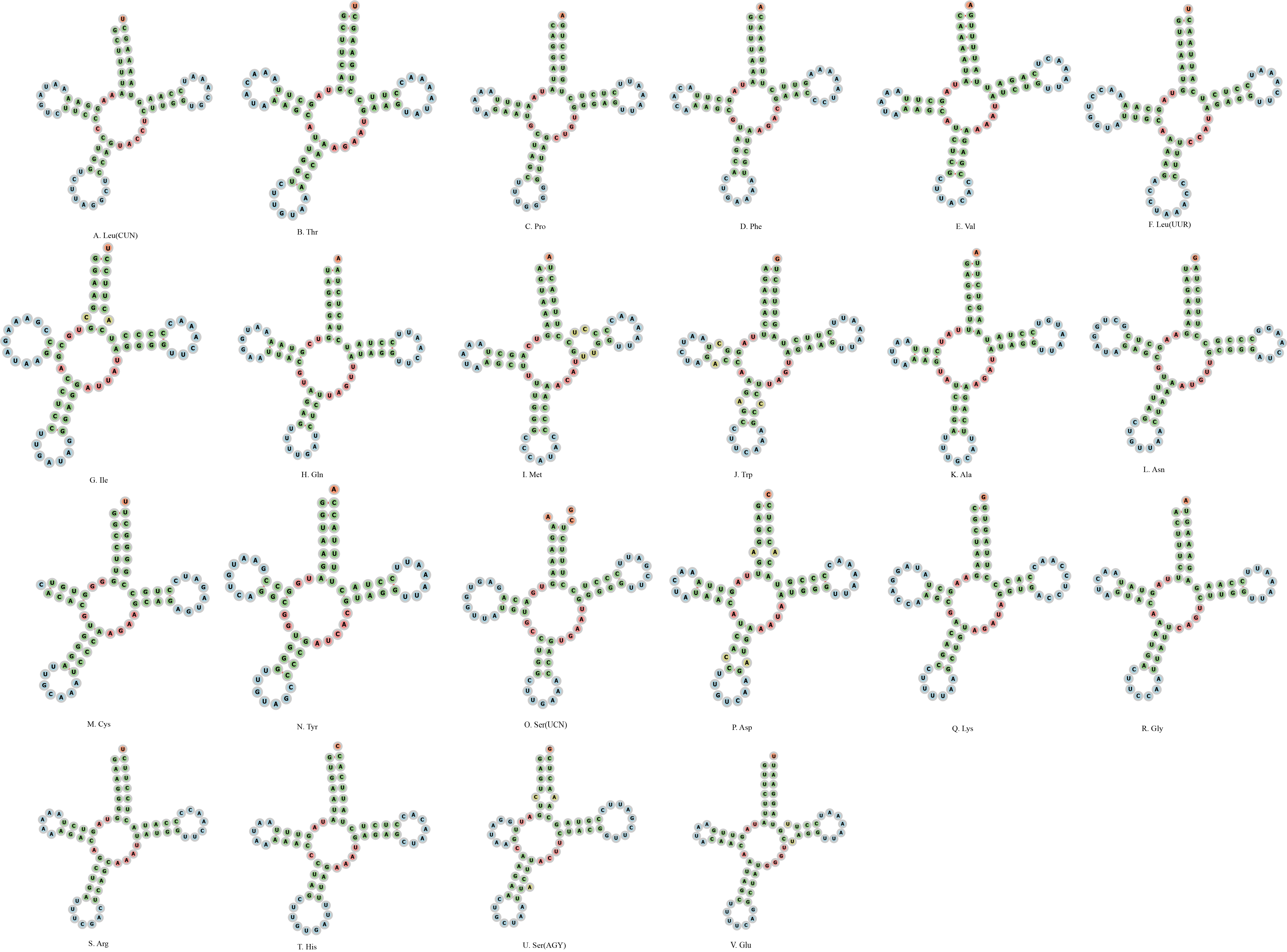

Supplement: Supplementary file 1 [file ijms-25-05967-s001.zip › Figure S3 GZAS.png]

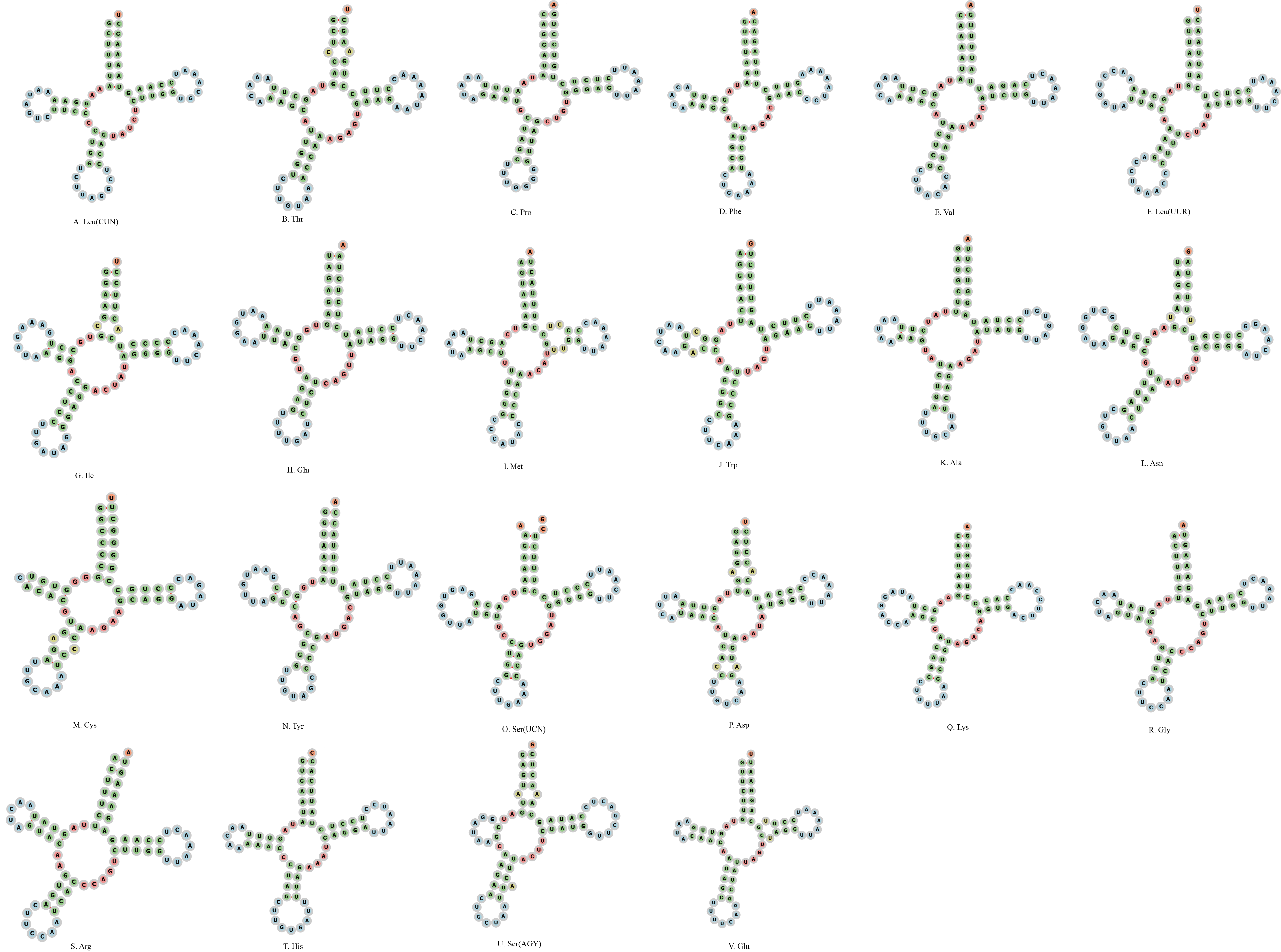

Supplement: Supplementary file 1 [file ijms-25-05967-s001.zip › Figure S4 RBYW.png]

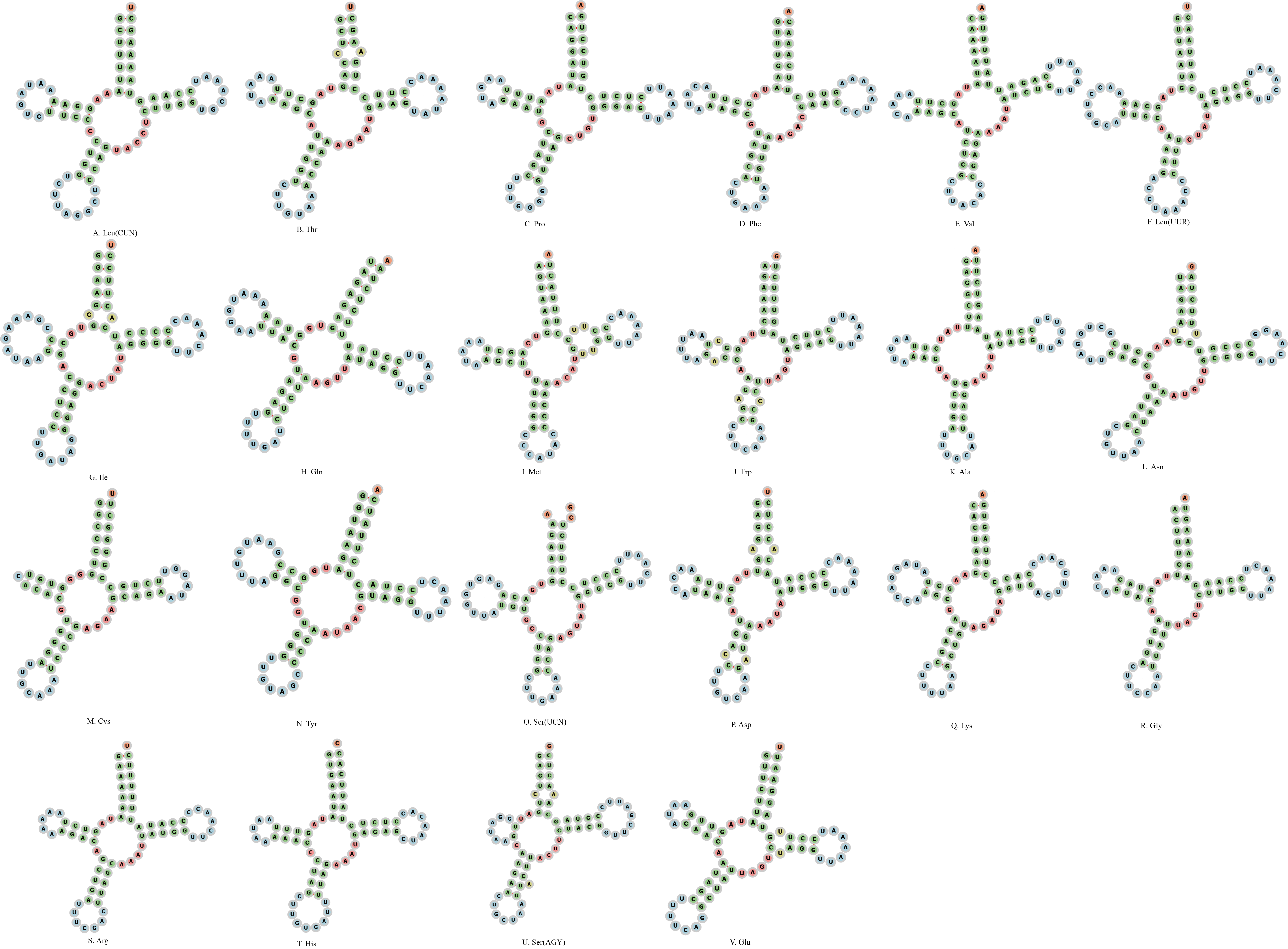

Supplement: Supplementary file 1 [file ijms-25-05967-s001.zip › Figure S5 GDMM.png]

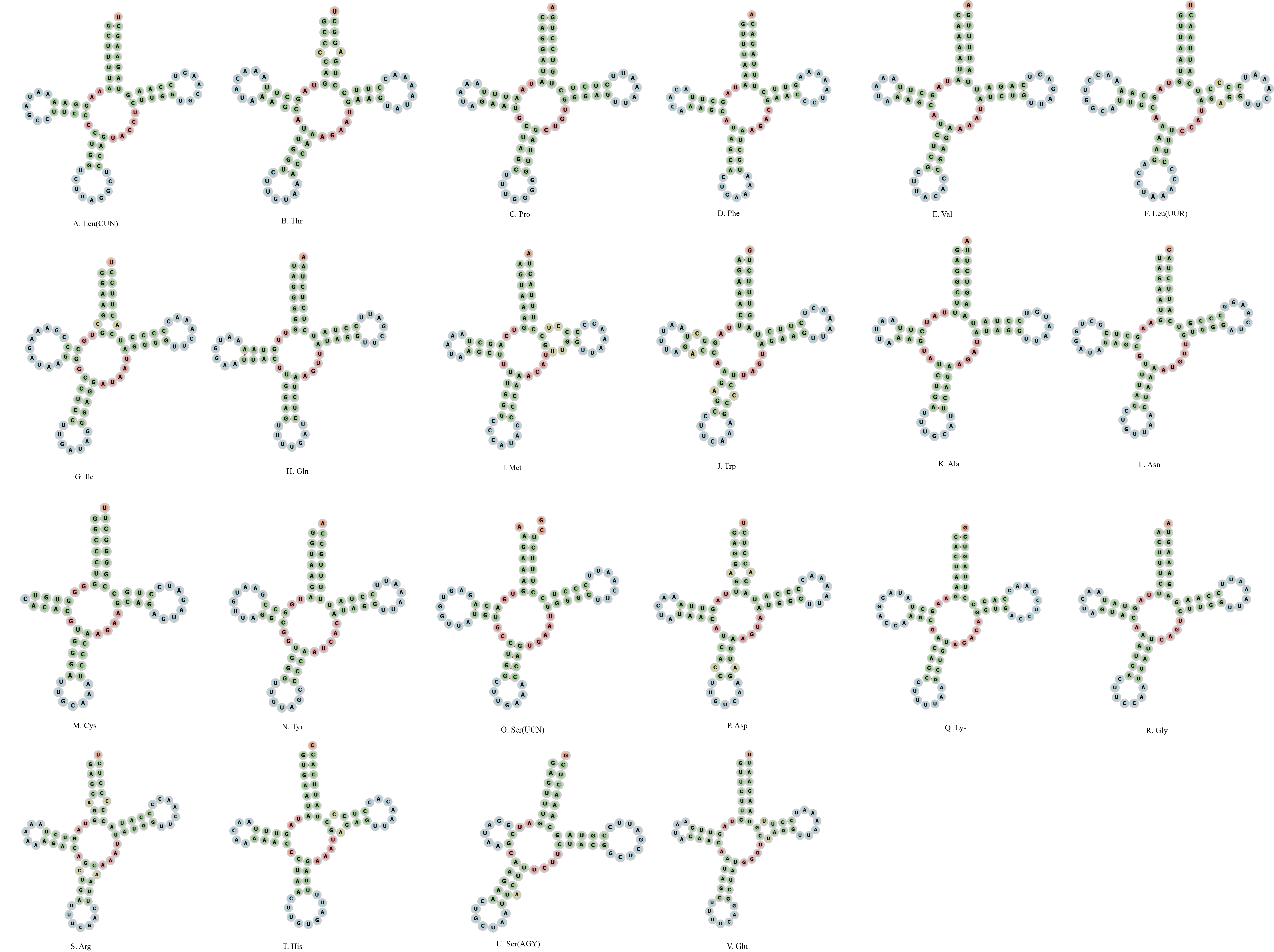

Supplement: Supplementary file 1 [file ijms-25-05967-s001.zip › Figure S6 SZWZ.png]

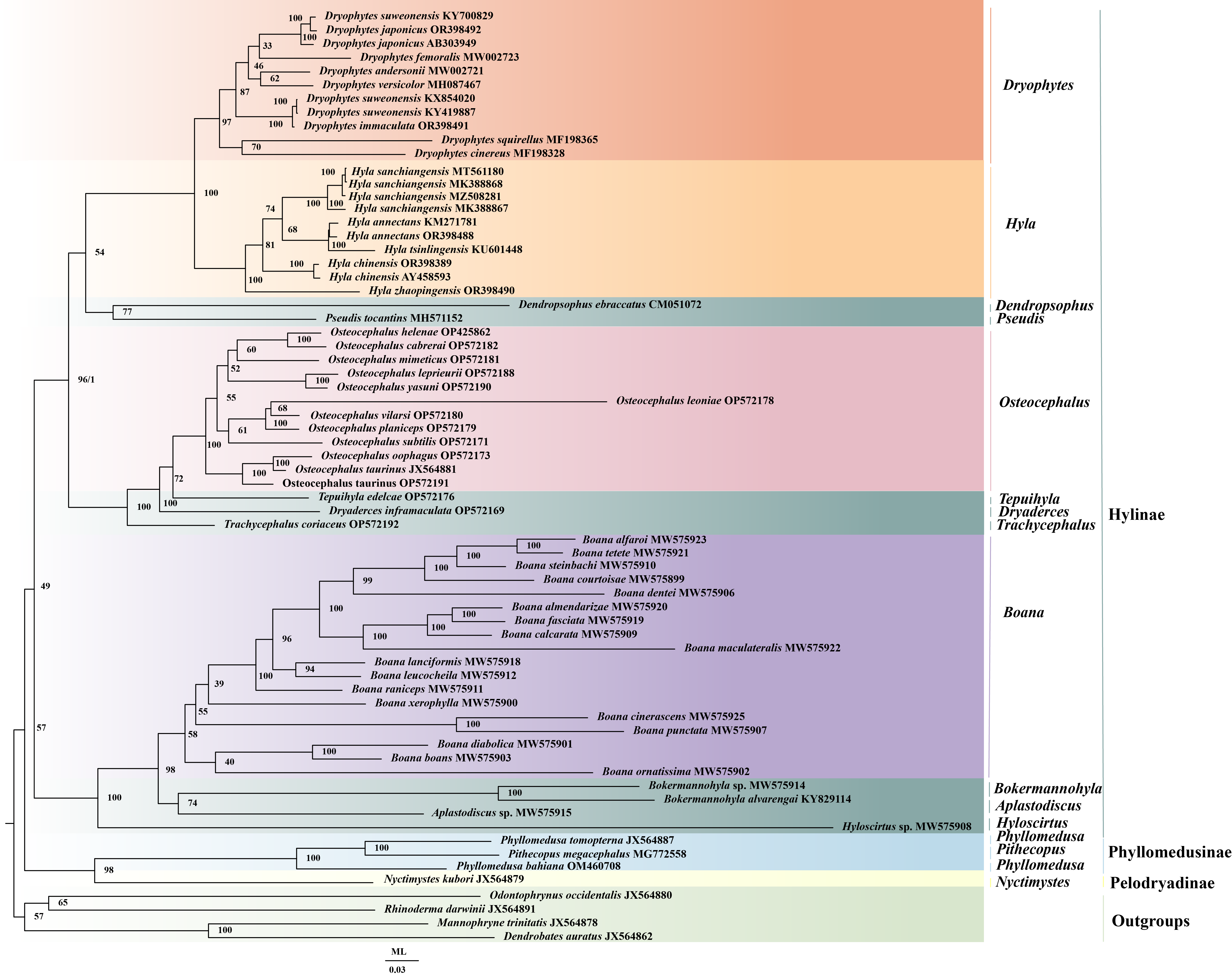

Supplement: Supplementary file 1 [file ijms-25-05967-s001.zip › Figure S7 12tree ML.png]

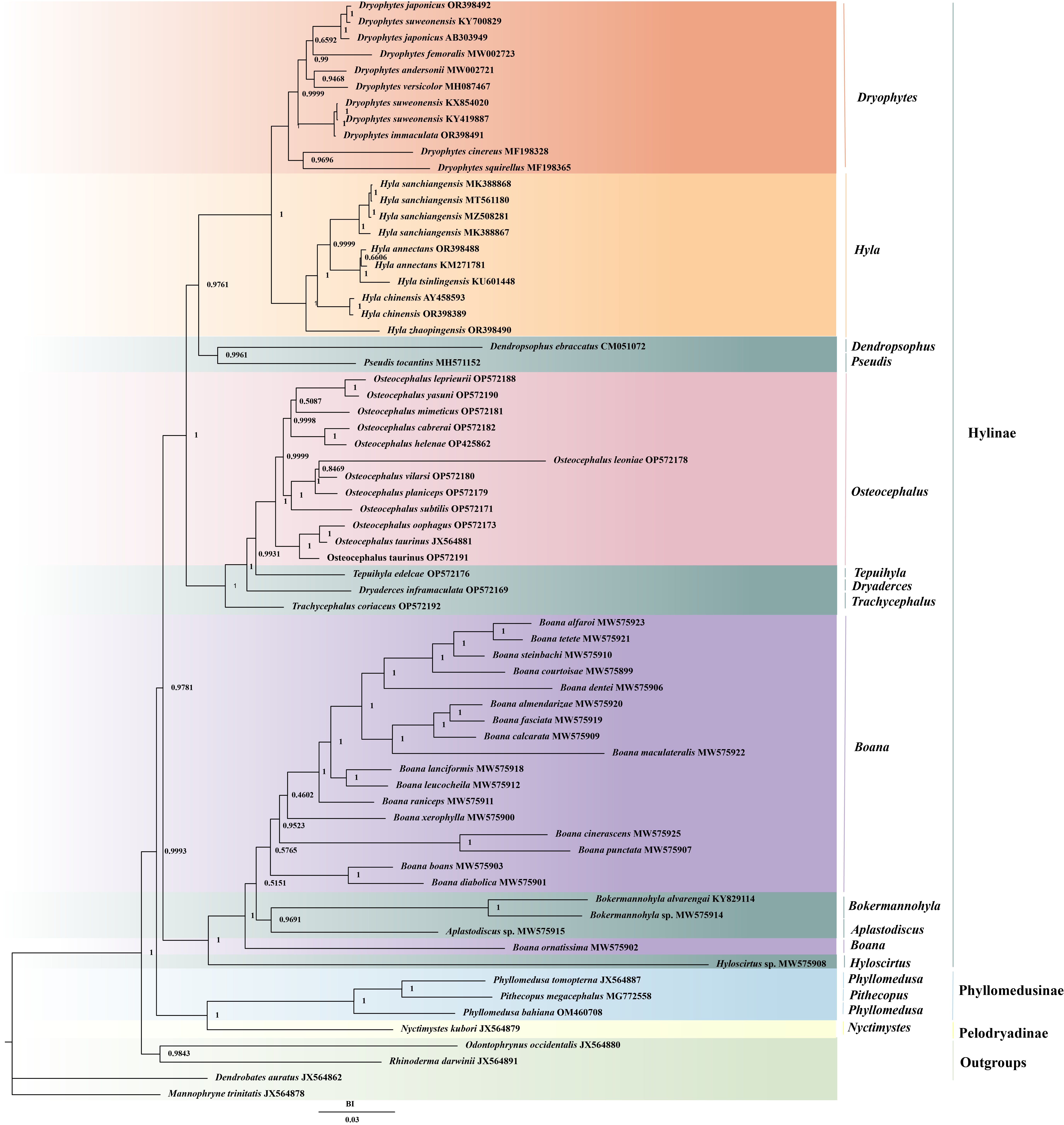

Supplement: Supplementary file 1 [file ijms-25-05967-s001.zip › Figure S8 12treeBI.png]
